# Impact of genomic stability on protein expression in endometrioid endometrial cancer

**DOI:** 10.1038/bjc.2012.67

**Published:** 2012-03-13

**Authors:** M I Lomnytska, S Becker, T Gemoll, C Lundgren, J Habermann, A Olsson, I Bodin, U Engström, U Hellman, K Hellman, A-C Hellström, S Andersson, M Mints, G Auer

**Affiliations:** 1 Department of Obstetrics and Gynaecology, Karolinska University Hospital, Solna, Karolinska Institute, SE-17176 Stockholm, Sweden; 2 Unit of Cancer Proteomics, Department of Oncology and Pathology, Karolinska Institute, Karolinska University Hospital, SE-17176 Stockholm, Sweden; 3 Department of Surgery, University of Lübeck, D-23538 Lübeck, Germany; 4 Department of Gynaecological Oncology, Radiumhemmet, Karolinska University Hospital, Solna, SE-17176 Stockholm, Sweden; 5 Department of Oncology and Pathology, Karolinska Institute, Karolinska University Hospital, SE-17176 Stockholm, Sweden; 6 Ludwig Institute for Cancer Research Ltd, Uppsala University, Box 595, Uppsala SE-75124, Sweden

**Keywords:** endometrioid endometrial cancer, genomic stability, marker protein patterns, atypical endometrioid hyperplasia

## Abstract

**Background:**

Genomic stability is one of the crucial prognostic factors for patients with endometrioid endometrial cancer (EEC). The impact of genomic stability on the tumour tissue proteome of EEC is not yet well established.

**Methods:**

Tissue lysates of EEC, squamous cervical cancer (SCC), normal endometrium and squamous cervical epithelium were subjected to two-dimensional (2D) gel electrophoresis and identification of proteins by MALDI TOF MS. Expression of selected proteins was analysed in independent samples by immunohistochemistry.

**Results:**

Diploid and aneuploid genomically unstable EEC displayed similar patterns of protein expression. This was in contrast to diploid stable EEC, which displayed a protein expression profile similar to normal endometrium. Approximately 10% of the differentially expressed proteins in EEC were specific for this type of cancer with differential expression of other proteins observed in other types of malignancy (e.g., SCC). Selected proteins differentially expressed in 2D gels of EEC were further analysed in an EEC precursor lesion, that is, atypical hyperplasia of endometrium, and showed increased expression of CLIC1, EIF4A1 and PRDX6 and decreased expression of ENO1, ANXA4, EMD and Ku70.

**Conclusion:**

Protein expression in diploid and aneuploid genomically unstable EEC is different from the expression profile of proteins in diploid genomically stable EEC. We showed that changes in expression of proteins typical for EEC could already be detected in precursor lesions, that is, atypical hyperplasia of endometrium, highlighting their clinical potential for improving early diagnostics of EEC.

Endometrial cancer (EC) is the fourth most common gynaecologic malignancy in Europe and Northern America. Even if detected at stage I, EC relapses in the majority of these cases (Creasman *et al*, 2006). Thus, diagnostics for detecting asymptomatic EC and precancer lesions is of paramount importance (Buchanan *et al*, 2009; Seebacher *et al*, 2009).

EC is divided into oestrogen-dependent endometrioid EC (EEC) that develops from atypical hyperplasia of endometrium (AH) and oestrogen-independent nonendometrioid EC that is usually characterised by a poorer prognosis (Bokhman, 1983; Horn *et al*, 2007). An important factor that defines the aggressiveness of malignancies, including EC, is chromosomal stability. More than half of the cases of EC are genomically stable and diploid (Lundgren *et al*, 2002, 2004). In comparison, all squamous cervical cancers (SCCs) and the vast majority of epithelial ovarian cancers are genomically unstable and aneuploid. Expression of proteins in diploid and aneuploid EC differs significantly (Lundgren *et al*, 2004). Characterisation of these proteins may provide new biomarkers for improved early diagnostics of EC and precursor lesions.

Proteomics is a potential method in the search for new cancer markers (Pitteri and Hanash, 2010; Sharon *et al*, 2010). Several proteomics-based studies of EC revealed important information about the endometrium, that is, the impact of genomic instability in endometrial cancer on protein expression (Lundgren *et al*, 2006), the proteome involved in myometrial invasion of endometrial cancer (Monge *et al*, 2009), and new insights into the secretome of endometrium (Casado-Vela *et al*, 2009). Unfortunately, only a few of the proteins identified in these studies were further analysed for their clinical value. Also, in many cases a comparison is only made between cancer and the respective normal tissue, without comparison with other closely related malignancies. Thus, the cancer specificity of the identified proteins could not be determined (Petrak *et al*, 2008). Furthermore, the similarities observed between protein expression in EEC and precursor lesions may be used for early detection of EEC. Finally, identification of proteins correlated with genomic instability has the potential to improve malignancy grading.

In the present study, we expand the current knowledge about the expression of proteins in EEC with respect to DNA ploidy as a measure of genomic stability and the relevance of these proteins to EEC carcinogenesis.

## Materials and methods

### Clinical material

Clinical material (Table 1a and b) was collected at the Department of Obstetrics and Gynaecology, Karolinska University Hospital, Huddinge; the Department of Gynaecologic Oncology, Radiumhemmet, Karolinska University Hospital, Solna, Sweden; and the Department of Oncology and Medical Radiology, Lviv National Medical University, Lviv, Ukraine, with informed consent and approval from the local ethics committees (Stockholm County Council – Dnr. 97-244 (1998-03-02), 00-068 (200-06-05), 2006/649-31/4, Ethics Committee of Lviv National Medical University – protocol no. 2).

Tissue biopsies of EEC (15 cases), SCC (13 cases) and control tissue from patients with nonmalignant gynaecological diseases (e.g., myoma and menorrhagia) consisting of normal endometrium (E; 8 cases) and squamous epithelium of cervical mucosa (SE; 4 cases) were collected before treatment for two-dimensional gel electrophoresis (2D; Table 1a). The tissue biopsies were snap frozen in liquid nitrogen and stored at −70 °C. Histopathological diagnosis was performed in all cases. Formalin-fixed paraffin-embedded (FFPE) tissue samples for immunohistochemical (IHC) analysis consisted of independent cases of EEC (19 cases), AH (15 cases) and normal endometrium (15 cases; Table 1b).

### DNA cytometry

**Figure 1:**
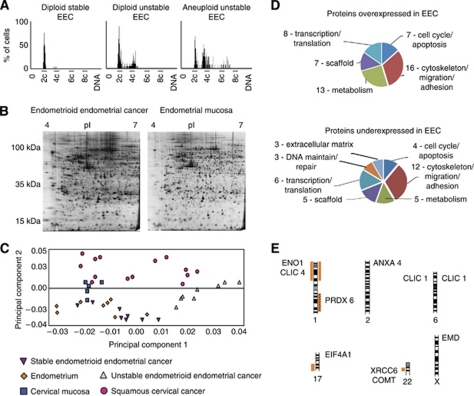
Description of the clinical material used in this study. (**A**) DNA histograms of diploid stable EEC showing narrow stem line in the 2c region, diploid unstable EEC with a broad stem line that expands from the 2c to the 4c region and aneuploid unstable EEC with a broad peak outside the 2c region and additional peaks exceeding the 4c region. (**B**) Examples of analysed 2D gels of EEC and endometrium. (**C**) Principal component analysis of the analysed 2D gels indicating similarity between the expression of protein spots in genomically unstable EEC and SCC, genomically stable EEC and normal endometrium as well as difference between the expression of protein spots in genomically stable and unstable EEC. (**D**) Clustering of identified proteins according to their function with numbers corresponding to the amount of detected proteins. (**E**) Distribution of selected proteins according to gains (to the right) and losses (to the left) on the chromosomes where the orange colour corresponds to early chromosomal changes during EEC carcinogenesis. A shaded pattern depicts chromosomal changes related to a bad prognosis for patients.

Tissue biopsies of EEC (Table 1a) and an independent group of FFPE samples of EEC and AH (Table 1b) were analysed for DNA ploidy. The former were analysed in imprint cytological samples and the latter in 6 *μ*m thick tissue cuts. The prepared slides were stained according to the Feulgen method and the DNA content in single cells was measured by means of image cytometry (Steinbeck *et al*, 1999). Histograms with a narrow stem line in the 2c region represented a diploid genomically stable subtype and those with a broad stem line in the 2c region that expanded towards the 4c region were classified as diploid genomically unstable (Figure 1A). Histograms with a narrow peak outside the 2c region were considered to be aneuploid genomically stable, whereas those with a broad peak outside the 2c region and additional peaks exceeding the 4c region were classified as aneuploid genomically unstable (Figure 1A).

### Two-dimensional gel electrophoresis and MALDI TOF mass spectrometry

Tissue proteins were extracted and solubilised in lysis buffer: 9 M urea (Bio-Rad, Sundbyberg, Sweden), 2 M thiourea (USB, Cleveland, OH, USA), 5% Resolyte (BDH, Poole, Dorset, UK), 65 mM DTT (Bio-Rad), 1 M EDTA (Merck, Darmstadt, Germany), 0.5% v/v Nonidet P-40 (USB), 25 mM CHAPS, 0.1% PMSF, 0.01% benzamidine, 0.01% BHT, and 35 mM NaOH (Sigma, St Louis, MO, USA) (Hellman *et al*, 2009). Protein concentration was determined using the Bradford protein assay (Bradford, 1976). The IEF, SDS–PAGE, staining with silver nitrate and excision of spots were performed as previously described (Lomnytska *et al*, 2010). Expression of protein spots was analysed by Progenesis SameSpot software (Nonlinear Dynamics, Newcastle upon Tyne, UK). Protein spots with a relative expression difference of 1.5-fold (ANOVA with *P*<0.05 and power >0.8) were selected for MALDI TOF MS. All steps were performed as previously described (Lomnytska *et al*, 2010).

### Western blot

**Figure 2:**
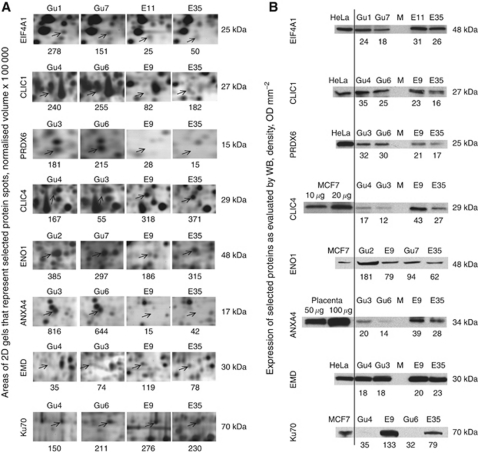
Expression of EIF4A1, CLIC1, PRDX6, CLIC4, ENO1, ANXA4, EMD, and Ku70 in 2D gels of endometrium, genomically stable EEC and genomically unstable EEC. (**A**) Selected areas of the 2D gels. Arrows indicate spots from which the selected proteins were identified. The numbers below indicate the normalised spot volume. Abbreviations: E=endometrium; Gu=genomically unstable endometrioid endometrial cancer. (**B**) Western blot (WB) analysis verifying protein expression patterns in the same samples as shown in (**B**). The numbers below the bands represent the densitometrical analysis.

In order to verify the identity of the proteins after MALDI TOF MS analysis, the same tissue protein lysates that were used for 2D gel analysis (Figure 2A) were subjected to western blot (Figure 2B). Equal concentration of protein lysates was applied to 10.5–14.0% SDS–PAGE (Criterion gels, Bio-Rad). The following commercial antibodies were used for western blot: EIF4A1 (1 : 2000; ab31217-100, rabbit polyclonal; Abcam, Cambridge, UK), CLIC1 (1 : 500; ab77214-100, mouse monoclonal; Abcam), PRDX6 (1 : 4000; ab59543, rabbit polyclonal; Abcam), CLIC4 (1 : 50; ab67593, rabbit polyclonal; Abcam), ENO1 (1 : 1000; ab85086, rabbit polyclonal; Abcam), ANXA4 (1 : 1000; ab109900, mouse monoclonal; Abcam), EMD (1 : 1000; ab54996, mouse monoclonal; Abcam) and Ku70 (1 : 1000; S5C11, mouse monoclonal; Abcam). All antibodies were diluted in Pierce (Rockford, IL, USA) Protein-Free T20 (PBS) Blocking Buffer (Thermo Scientific, Middletown, VA, USA) and incubated for 12 h at 4 °C. As positive controls, lysates of cell lines that contain corresponding antigens were used, that is, HeLa cell lysate for EIF4A1, CLIC1, PRDX6 and EMD, placenta lysate for ANXA4 and MCF7 cell lysate for CLIC4, ENO1 and Ku70 (Figure 2B). The membranes were incubated in secondary antibody of the corresponding species, diluted 1 : 15 000 in Pierce Protein-Free T20 (PBS) Blocking Buffer for 1.5 h at room temperature, followed by 4 washes of 15 min in PBS-T. Finally, the proteins were visualised by ECL. The secondary antibodies used were HRP-linked anti-mouse (NXA931) and HRP-linked anti-rabbit antibodies (NA934VS, GE Healthcare, Chalfont St Giles, UK). All steps were performed as described before (Lomnytska *et al*, 2010).

### Immunohistochemistry

**Figure 3:**
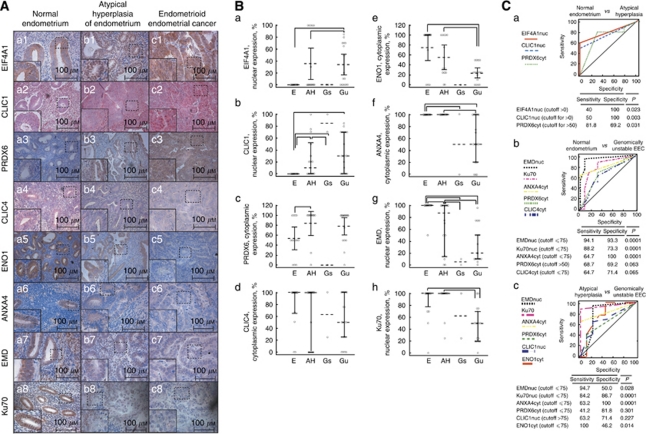
Analysis of the expression of EIF4A1, CLIC1, PRDX6, CLIC4, ENO1, ANXA4, EMD and Ku70. (**A**) Examples of the immune staining in endometrium (a), atypical hyperplasia of endometrium (b) and endometrioid endometrial cancer (c). Inserts indicate an × 400 magnification of the indicated areas. (**B**) Comparison between expression of proteins (panels a–h) in endometrium (15 cases), atypical hyperplasia of endometrium (15 cases), genomically stable endometrioid endometrial cancer (2 cases) and genomically unstable endometrioid endometrial cancer (17 cases) as evaluated by immunohistochemistry. Horizontal lines indicate statistically significant differences between the protein expression in compared groups (ANOVA, Kruskall–Wallis, *P*<0.05). Abbreviations: AH=atypical hyperplasia of endometrium; E=endometrium; Gs=genomically stable endometrioid endometrial cancer; Gu=genomically unstable endometrioid endometrial cancer. (**C**) Sensitivity and specificity for discrimination between (a) endometrium and atypical hyperplasia of endometrium, (b) endometrium and genomically unstable endometrioid endometrial cancer, and (c) atypical hyperplasia of endometrium and genomically unstable endometrioid endometrial cancer as evaluated by receiver-operator curves.

An immunohistochemical analysis was carried out on FFPE samples of EEC, AH and E of an independent group of patients in order to study the expression of the identified proteins during EEC carcinogenesis (Table 1b). Immunohistochemistry was performed using the two-step streptavidin–biotin method. Tissue slides were incubated overnight with the primary antibodies in 1% BSA at 4 °C. Antibodies used previously for western blot were applied in following dilutions for IHC: EIF4A1 (1 : 200), CLIC1 (1 : 10), PRDX6 (1 : 1000), ENO1 (1 : 200) and Ku70 (1 : 400). In addition, staining against ANXA4 (1 : 200; sc-1930, goat polyclonal; Santa Cruz Biotechnology, Santa Cruz, CA, USA), CLIC4 (1 : 30; HPA008019, rabbit polyclonal; Sigma-Aldrich, St Louis, MO, USA) and EMD (1 : 3000; HPA000609, rabbit polyclonal; Sigma-Aldrich) was performed (Figure 3A). Antibodies used for western blot against ANXA4, CLIC4 and EMD were also used for IHC for confirmation of specificity (data not shown). Several visualising systems were used: VectaStain (Vector, Peterborough, UK) ABC-Po-kit and DAB (positive stain was brown), LSAB+ (DAKO, Glostrup, Denmark) (positive stain was red). Control tissues that contained corresponding antigens were also utilised: placenta tissue for ANXA4, placenta and tonsillar tissue for EIF4A1, tonsillar and ovarian tissue for CLIC1, tonsillar and placenta tissue for PRDX6, tonsillar, placenta and breast cancer tissue for CLIC4, colon cancer and tonsillar tissue for EMD, breast cancer and tonsillar tissue for Ku70 and breast cancer and kidney tissue for ENO1. Images were captured with a Leica DM4500B (camera DFC320, ocular 10 × , objectives 20 × /0.50 HC PL and 40 × , 506145) and the Leica Application Suite software, version 2.4.0 (Wetzlar, Germany) as 16-bit depth .tif format images with 48-bit image resolution, and expression of the analysed proteins was scored as previously described (Cheng *et al*, 2008; Lomnytska *et al*, 2011).

### Statistical analysis

We used the inbuilt statistical chapter of SameSpot Nonlinear software (PCA, ANOVA, power, *t*-test), MedCalc, version 11.1.1.0 (Mariakerke, Belgium) (receiver-operator-characteristic (ROC) curves) and Statistica 6.0 (Tulsa, OK, USA), (correlation, *t*-test, *χ*^2^ test). A difference of *P*<0.05 was considered statistically significant.

## Results

### Expression of protein spots in analysed 2D gels

A total of 42 2D gels were generated from tissue biopsies of 40 patients with EEC, SCC or nonmalignant gynaecological diseases (Table 1a), with each gel containing ∼2000 protein spots (Figure 1B). DNA cytometry was performed on all EEC samples in order to identify their genomic stability (Table 1a and Figure 1A).

Based on the DNA pattern, EEC cases were divided into two major groups – genomically stable EEC that included five diploid stable cases and two aneuploid stable cases and genomically unstable EEC that consisted of seven diploid unstable cases and one aneuploid unstable case. We performed a principal component analysis (PCA) that considered expression of all protein spots in a 2D gel (Figure 1C). Squamous cervical cancer was included in the comparison as a discriminative cancer with a different pathogenesis and that is characterised by genomic instability. According to the analysis, genomically stable EEC (7 cases), genomically unstable EEC (8 cases), SCC (13 cases), normal endometrium (8 cases) and squamous cervical mucosa (4 cases) clustered separately. Some proximity was observed between the genomically unstable EEC and SCC and between the genomically stable EEC and normal endometrium (Figure 1C).

We identified 121 differentially expressed proteins (Tables 2 and 3 and Supplementary Table S1). The majority of the proteins were overexpressed in the studied cancers. By comparison of EEC and SCC, we extracted 12 proteins explicitly overexpressed in genomically unstable EEC (Tables 2 and 3). Proteins overexpressed in EEC included those that were more expressed in genomically unstable EEC than in genomically stable EEC (44 proteins) and proteins that were more expressed in genomically unstable EEC than in SCC (29 proteins). We did not identify any proteins that were overexpressed in genomically stable EEC in comparison with normal endometrium. Only a relative overexpression of 27 proteins in genomically stable EEC was observed in comparison with genomically unstable EEC and SCC (Tables 2 and 3 and Supplementary Table S1).

### Function relevance of the identified proteins

Functional activity of the identified proteins was analysed using the NCBI/Protein and OMIM databases. We divided the proteins in the major functional groups, that is, regulators of cell cycle and apoptosis, migration and adhesion, metabolism, transcription and translation, maintainers of DNA, members of extracellular matrix and scaffold proteins. We observed that the representation of the proteins that were over- or under-expressed in EEC in the studied functional groups was unequal (*P*=0.0006; Figure 1D).

### Verification of protein identification

In order to confirm the accuracy of the protein identification with MALDI TOF MS, the tissue protein lysates used for 2D gels were immunoblotted using commercially available antibodies against eight selected proteins: EIF4A1, CLIC1, PRDX6, CLIC4, ENO1, ANXA4, EMD and Ku70 (Figure 2). Expression of the protein spots in 2D gels of two selected cases of EEC and two controls is shown (Figure 2A). Concomitantly, their expression was verified using western blot for these respective cases (Figure 2B), confirming their expression profile in EEC. The expression profile of EIF4A1 and ANXA4 could not be confirmed by western blot but, conversely, on 2D gels their observed molecular weight was lower than expected. This could be because of cancer-specific overexpression of truncated forms of these proteins.

### Expression of EIF4A1, CLIC1, PRDX6, CLIC4, ENO1, ANXA4, EMD and Ku70 in genomically stable and unstable EEC, AH and endometrium as evaluated by IHC

As these identified proteins have not been previously analysed in connection with EEC, their expression was investigated in greater detail. Therefore, a set of independent cases was subjected to IHC, encompassing normal endometrium, AH, a precursor lesion of EEC and genomically stable and unstable EEC (Table 1b). According to the IHC analysis, expression of EIF4A1 and CLIC1 increased in the nuclei of atypical cells and cytoplasmic expression of PRDX6 was enhanced in AH and genomically unstable EEC. A tendency towards decreased cytoplasmic expression of CLIC4 was observed in genomically stable and unstable EEC. Although ENO1 was not significantly overexpressed in 2D gels of EEC (Table 3 and Figure 2A), its cytoplasmic expression was low in AH and genomically unstable EEC (Figure 3Be). Also, low cytoplasmic expression of ANXA4 was observed in genomically stable and unstable EEC (Figure 3Bf). Interestingly, only the N-terminal part of ANXA4 was significantly overexpressed in 2D gels of EEC. This fragment migrated at 17 kDa whereas the molecular mass of the full-length protein is 34 kDa (Figure 2). Nuclear expression of EMD was low in AH, genomically stable and unstable EEC. Expression of Ku70 was highly abundant in endometrium and low in genomically stable and unstable EEC (Figure 3A and B).

Using ROC curves (Figure 3C), we determined that the expression of CLIC1, EIF4A1 and PRDX6 displayed the highest sensitivity and specificity for discrimination between E and AH (Figure 3Ca). Expression of EMD, Ku70 and ANXA4 depicted the highest sensitivity and specificity for discrimination between E, AH and genomically unstable EEC (Figure 3Cb and c). Thus, we demonstrated that changes in protein expression observed in EEC can already be detected on the level of AH. No statistically significant difference was found between the expression of the proteins in genomically stable and unstable AH.

## Discussion

Malignancies are classically divided into diploid and aneuploid based on DNA ploidy. However, it has been shown in breast cancer that further subclassification into stable and unstable diploid and aneuploid tumours provides more accurate prognosis (Kronenwett *et al*, 2006). Our analysis of the tissue proteome of EEC offered a possibility for re-classification of this malignancy into stable and unstable subtypes. In particular, our analysis of 2D gels did not show any difference between the expression of proteins in diploid and aneuploid genomically unstable EEC, but showed a clear difference with diploid genomically stable EEC. In addition, similarities were observed between protein expression in genomically unstable SCC and genomically unstable ECC, suggesting an impact of genomic instability on protein expression. By comparing EEC and SCC, we identified changes in protein expression specific for EEC while excluding proteins commonly overexpressed in most malignancies (Petrak *et al*, 2008).

We also confirmed the identity of several proteins previously found to be overexpressed in endometrial cancer. One interesting example was CAPS (Li *et al*, 2008a), a protein related to low differentiation and worse survival of patients with endometrial cancer (Li *et al*, 2008b). Among the proteins linked to proliferation and invasion of endometrial cancer (Yi *et al*, 2009), we identified HSPA1, TPM2, PDIA, ENO and HNRNPK. Among the proteins downregulated in EEC in connection to invasion into myometrium (Monge *et al*, 2009), we identified MSN (family of EZR), TUBA1B, ANXA1, HNRNPH3 and TALDO1. We also observed a high expression of HSP90AA1, PTGES3 and ATP5B in relation to the stage of EEC (Supplementary Table S2).

Our study focussed on the analysis of protein expression in EEC whereas other groups have analysed chromosomal changes in EEC and in AH (Sonoda *et al*, 1997; Suzuki *et al*, 1997; Kiechle *et al*, 2000; Baloglu *et al*, 2001; Schulten *et al*, 2004; Levan *et al*, 2006; O’Toole *et al*, 2006) and CIN3 and SCC (Heselmeyer *et al*, 1996, 1997) (Supplementary Table S3 and Figure 1E). Once synthesised, proteins generally undergo numerous post-translational modifications in order to become functionally active. We observed underexpression of ENO1 and CLIC4 in both EEC and AH. Interestingly, loss of a specific part of the 1p chromosome is a key event during EEC carcinogenesis and this deleted region is responsible for the synthesis of ENO1 and CLIC4 (Kiechle *et al*, 2000; Baloglu *et al*, 2001). Other early events during EEC carcinogenesis are gains in the entire long arm of the 1q chromosome that contains the gene coding for PRDX6 and losses at 22q chromosome that disrupt the synthesis of Ku70 (XRCC6) (Kiechle *et al*, 2000; Baloglu *et al*, 2001), which also corresponds to our findings on the protein level in EEC and AH. In addition, EEC is characterised by gains at the 2p, 6p, 17p and Xq chromosomes (Suzuki *et al*, 1997) and those are responsible for the synthesis of ANXA4, CLIC1, EIF4A1 and EMD, respectively. In contrast to this, we observed decreased expression of ANXA4 in AH and EEC according to our IHC data, whereas we confirmed increased expression on our 2D gels. This discrepancy can be explained by the fact that the molecular weight of ANXA4 detected on the 2D gels was lower than expected and the protein was represented only by the NH2 domain. This can be due to cancer-specific truncation of the NH2 domain, leading to malfunction of the full-length protein (Gerke and Moss, 2002). EMD was also underexpressed in EEC and AH, which corresponds to its functional role in maintaining chromosomal stability.

For the first time, our paper describes EIF4A1, CLIC1, PRDX6, CLIC4, ENO1, ANXA4, EMD and Ku70 in relation to EEC, although their role is well established in other cancers. *EIF4A1* is overexpressed in hepatocellular carcinoma (Yoon *et al*, 2006) and is an early marker of distant metastases of non-small cell lung cancer (Ji *et al*, 2003). Similarly, we find it overexpressed in AH, suggesting that EIF4A1 expression could also be used as an early marker of EEC. *CLIC1* is involved in invasion, cancer cell motility (Wang *et al*, 2009) and development of chemoresistance (Kang and Kang, 2008). It is overexpressed in nasopharyngeal carcinoma (Chang *et al*, 2009), colorectal cancer (Petrova *et al*, 2008) and hepatocellular cancer (Huang *et al*, 2004). *PRDX6* protects against oxidative injury, it is overexpressed in endometriosis (Stephens *et al*, 2010) and it increases the invasiveness of breast cancer (Chang *et al*, 2007). *CLIC4* is a chloride intracellular channel that translocates to the nucleus in response to DNA damage and is associated with growth arrest and apoptosis. Moreover, loss of the expression of CLIC4 in cells and upregulation in stroma is associated with malignant progression (Suh *et al*, 2007a,2007b). *ENO1* is a glycolytic enzyme that binds to the promoter of the oncogene c-myc and acts as a transcriptional repressor (Feo *et al*, 2000). Therefore, we hypothesise that loss of ENO1 leads to increased c-myc expression, which is known to promote carcinogenesis. The transcription and translation of *ANXA4* in endometrium is regulated by progesterone, an important regulator of cyclic changes in endometrium (Ponnampalam and Rogers, 2006). *EMD* belongs to the inner nuclear membrane proteins that bind chromatin modifiers (Shaklai *et al*, 2007). Its loss in ovarian cancer is considered to be the basis for aneuploidy (Capochichi *et al*, 2009). *Ku70*, or *XRCC6*, is a nuclear complex involved in the repair of double-strand non-homologous DNA breaks. Malfunction of the *XRCC6* gene is observed in ovarian cancer (Kim *et al*, 2010) and breast cancer (Willems *et al*, 2009).

In summary, we analysed the tissue proteome of EEC with respect to genomic stability, one of the most important prognostic markers (Lundgren *et al*, 2002, 2004), and identified differentially expressed proteins. We showed that changes in protein expression could already be detected in precursor lesions, that is, atypical hyperplasia of endometrium, which could provide significant improvement in early detection of EEC.

## Figures and Tables

**Table 1:** Description of clinical material used for (a) 2D gel electrophoresis and (b) immunohistochemical analysis

**(a)**					
**No.**	**Sample ID**	**TNM**	**Stage, FIGO, 1988**	**Ploidy**	**Age**
*I. Endometrioid endometrial cancer*					
*I.I.* *Genomically stable*					
1	Gs1	T1aN0G1	IA	DS	54
2	Gs2	T1aN0G2	IA	DS	82
3	Gs3	T1aN0G2	IA	AS	51
4	Gs4	T1bN0G1	IB	DS	69
5	Gs5	T1bN0G1	IB	DS	86
6	Gs6	T1bN0G1	IB	AS	84
7	Gs7	T1cN0G3	IC	DS	69
				70.7±14.2	
					
*I.II.* *Genomically unstable*					
8	Gu1	T1bN0G1	IB	DU	85
9	Gu2	T1bN0G1	IB	DU	52
10	Gu3	T1bN0G1	IB	DU	80
11	Gu4	T1bN0G2	IB	DU	52
12	Gu5	T1cN0G1	IC	DU	41
13	Gu6	T1cN0G1	IC	DU	79
14	Gu7	T1cN0G2	IC	AU	71
15	Gu8	T3N1G3	III	DU	54
				64.3±16.4	
			**Stage, FIGO, 1994**		
*All:*				67.3±15.2	
*II. Squamous cervical cancer*					
16	CC1	T1b1N0G2	IB1		65
17	CC2	T1bN0G3	IB1		52
18	CC3	T1b2N0G2	IB2		45
19	CC4	T1b2N0G2	IB2		39
20	CC5	T1b2N0G2	IB2		59
21	CC6	T1b2N0G3	IB2		53
22	CC7	T2aN0G2	IIA		69
23	CC8	T2aN0G2	IIA		44
24	CC9	T2aN0G3	IIA		89
25	CC10	T2aN0G3	IIA		63
26	CC11	T2bN0G3	IIB		45
27	CC12	T3N0G3	III		60
28	CC13^a^	T1b2N0	IB2		41
				55.7±14.0	
*III. Endometrium*					
	E9, E10, E11, E13, E13, E16, E29, E35			50.6±2.7	
*IV. Cervical mucosa*					
	M1, M2, M4, M5			49.5±7.1	
					
**(b)**					
**No.**	**Age**	**TNM**	**Stage, FIGO, 1988**	**Relapse, months**	**Overall survival, months**
*Endometrioid endometrial cancer*					
*Diploid stable,* n*=2*					
2	78	T1bNxM0G1	IB	—	72
3	62	T1cNxM0G1	IC	—	72
					
*Diploid unstable,* n*=13*					
1	47	T1bNxM0G2	IB	—	64
4	73	T1bNxM0G1	IB	—	58
5	75	T3NxM1G3	IV	38 (distant)	70
6	72	T1bNxM0G1	IB	—	72
7	78	T1bNxM0G1	IB	10 (local)	72
8	71	T1aNxM0G1	IA	—	72
9	71	T1cNxM0G1	IC	18 (distant)	33
10	65	T1bNxM0G2	IB	—	72
11	63	T1cNxM0G3	IC	—	72
12	58	T1bNxM0G2	IB	—	72
13	57	T3aNxM0G2	IIIA	—	72
14	58	T1aNxM0G1	IA	—	72
15	63	T1bNxM0G1	IB	—	72
					
*Aneuploid unstable,* n*=4*					
16	82	T1cNxM0G3	IC	22 (local)	24
17	75	T1bNxM0G3	IB	10 (distant)	38
18	58	T2bNxM0G3	IIB	—	72
19	54	T1bNxM0G1	IB	—	72
66.5±9.3					
Atypical hyperplasia of endometrium					
60.8±10.9		Diploid unstable, *n*=8			
		Diploid stable, *n*=7			
Normal endometrium					
50.1±3.7		*n*=15			

Abbreviations: 2D=two dimensional; AS=aneuploid stable; AU=aneuploid unstable; DS=diploid stable; DU=diploid unstable; FIGO=International Federation of Gynaecology and Obstetrics (Fédération Internationale de Gynécologie et d'Obstétrique); Gs=genomically stable endometrioid endometrial cancer; Gu=genomically unstable endometrioid endometrial cancer; TNM=Tumour, Node, and Metastasis.

a Adenocarcinoma of cervix uteri. Underlined entries for overall survival correspond to deceased patients.

**Table 2:**
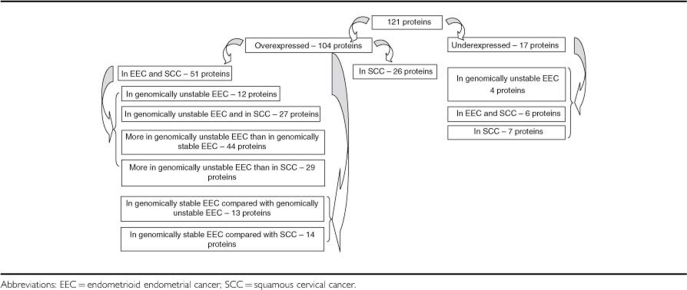
Overview of the expression of identified proteins

**Table 3:**
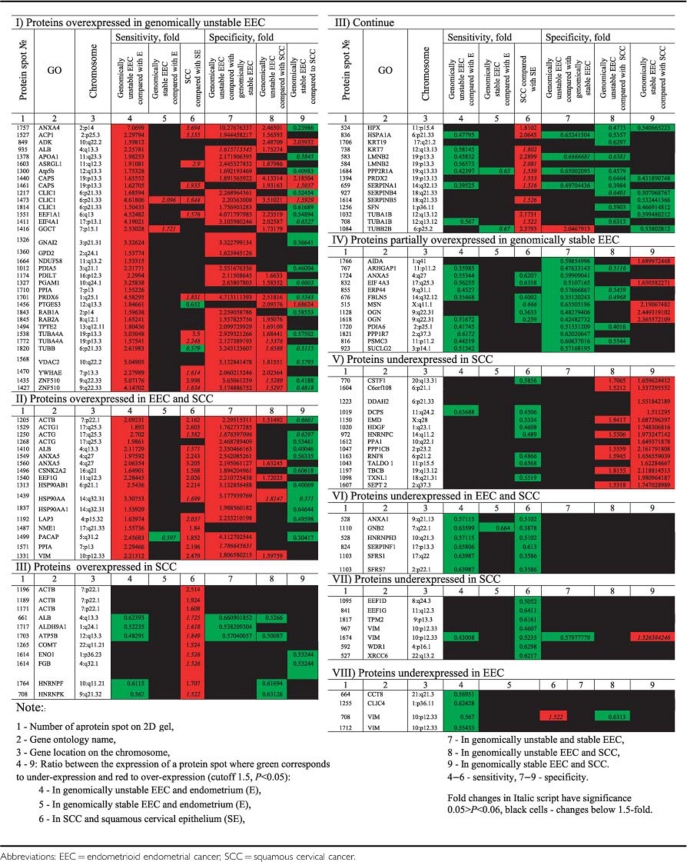
Expression of identified proteins in genomically stable and unstable EEC in comparison with SCC
