# Supplementary material for: Impact of genomic stability on protein expression in endometrioid endometrial cancer
Source: Br J Cancer. 2012 Mar 13;106(7):1297–305. doi: 10.1038/bjc.2012.67 (PMC3314786; doi:10.1038/bjc.2012.67)
Supplement: Supplementary Table 1 [file bjc201267x1.doc]

| Supplementary table 1. Description of identified proteins | | | | | | | | | | | | | | | | | | | | | | | | | | | | | | |
| --- | --- | --- | --- | --- | --- | --- | --- | --- | --- | --- | --- | --- | --- | --- | --- | --- | --- | --- | --- | --- | --- | --- | --- | --- | --- | --- | --- | --- | --- | --- |
| Protein spot № | Protein identity | | GO | | | Chromosome | | | Empirical | | | | | In silica | | | | | Accession number, NCBI | | | Sequence coverage, % | | | Matched peptides | | | Total amount of peptides | | |
|  |  |  |  |  |  |  |  |  | pI | MW, kDa | | | | pI | | | MW, kDa | |  |  |  |  |  |  |  |  |  |  |  |  |
| 1 | 2 | | 3 | | | 4 | | | 5 | 6 | | | | 7 | | | 8 | | 9 | | | 10 | | | 11 | | | 12 | | |
| 1527 | Acid phosphatase 1, soluble iso c | | ACP1 | | | 2:p25.3 | | | 6.5 | 15 | | | | 6 | | | 18.84 | | NP_004291.1 | | | 48 | | | 6 | | | 37 | | |
| 1171 | ACTB | | ACTB | | | 7:p22.1 | | | 5 | 25 | | | | 6 | | | 40.5 | | AAH12854.1 | | | 14 | | | 4 | | | 29 | | |
| 1189 | ACTB | | ACTB | | | 7:p22.1 | | | 4.5 | 30 | | | | 6 | | | 40.5 | | AAH08633 | | | 23 | | | 6 | | | 40 | | |
| 1196 | ACTB | | ACTB | | | 7:p22.1 | | | 5 | 30 | | | | 6 | | | 40.5 | | AAH12854.1 | | | 27 | | | 6 | | | 41 | | |
| 1205 | ACTB | | ACTB | | | 7:p22.1 | | | 4.5 | 30 | | | | 6 | | | 40.54 | | AAH12854.1 | | | 27 | | | 6 | | | 53 | | |
| 1529 | ACTG1 protein | | ACTG1 | | | 17:q25.3 | | | 4.7 | 15 | | | | 5 | | | 18.72 | | AAH10417.2 | | | 48 | | | 4 | | | 28 | | |
| 1250 | Actin, gamma | | ACTG | | | 17:q25.3 | | | 5.5 | 30 | | | | 6 | | | 26 | | AA51580 | | | 28 | | | 6 | | | 55 | | |
| 1268 | Actin, gamma | | ACTG | | | 17:q25.3 | | | 5 | 25 | | | | 6 | | | 26 | | AAA51580 | | | 26 | | | 3 | | | 37 | | |
| 849 | Adenosyne kinase | | ADK | | | 10:q22.2 | | | 6.5 | 45 | | | | 6 | | | 39 | | NP_001114 | | | 11 | | | 5 | | | 40 | | |
| 661 | Albumin | | ALB | | | 4:q13.3 | | | 6 | 60 | | | | 6 | | | 58.53 | | [AAG35503.1](http://www.ncbi.nlm.nih.gov/entrez/viewer.fcgi?db=protein&val=11493459) | | | 53 | | | 26 | | | 81 | | |
| 935 | Albumin | | ALB | | | 4:q13.3 | | | 4 | 40 | | | | 6 | | | 71.35 | | NP_000468.1 | | | 14 | | | 6 | | | 35 | | |
| 1410 | Albumin | | ALB | | | 4:q13.3 | | | 5.5 | 20 | | | | 6 | | | 27.2 | | 1TF0 A | | | 23 | | | 16 | | | 35 | | |
| 659 | Alpha-1-antitrypsin | | SERPINA1 | | | 14:q32.13 | | | 4.7 | 50 | | | | 5 | | | 44.32 | | [1HP7 A](http://www.ncbi.nlm.nih.gov/entrez/viewer.fcgi?db=protein&val=13787109) | | | 38 | | | 15 | | | 88 | | |
| 708 | Alpha-tubulin | | TUBA1B | | | 12:q13.12 | | | 5.5 | 50 | | | | 5 | | | 50.82 | | [AAA91576.1](http://www.ncbi.nlm.nih.gov/entrez/viewer.fcgi?db=protein&val=340021) | | | 27 | | | 7 | | | 56 | | |
| 1548 | Alpha-tubulin | | TUBA4A | | | 19:p13.3 | | | 4.3 | 15 | | | | 5 | | | 50.5 | | CAA30026.1 | | | 15 | | | 5 | | | 46 | | |
| 1549 | Annexin 5, C- | | ANXA5 | | | 4:q27 | | | 4.5 | 15 | | | | 5 | | | 36 | | NP_001145.1 | | | 21 | | | 7 | | | 41 | | |
| 1560 | Annexin 5, N- | | ANXA5 | | | 4:q27 | | | 4.5 | 15 | | | | 5 | | | 36 | | NP_001145.1 | | | 25 | | | 6 | | | 25 | | |
| 528 | Annexin 1 | | ANXA1 | | | 9:q21.13 | | | 6.5 | 70 | | | | 7 | | | 38.92 | | [NP_000691.1](http://www.ncbi.nlm.nih.gov/entrez/viewer.fcgi?db=protein&val=4502101) | | | 27 | | | 7 | | | 49 | | |
| 1757 | Annexin 4 | | ANXA4 | | | 2:p14 | | | 6.7 | 17 | | | | 6 | | | 33.76 | | AAH63672.1 | | | 17 | | | 6 | | | 75 | | |
| 1724 | Annexin 5 | | ANXA5 | | | 4:q27 | | | 5 | 33 | | | | 5 | | | 35.8 | | NP_001145 | | | 49 | | | 17 | | | 55 | | |
| 1378 | Apolipoprotein A1 | | APOA1 | | | 11:q23.3 | | | 5 | 25 | | | | 5 | | | 28.94 | | AAA51747.1 | | | 37 | | | 10 | | | 49 | | |
| 1603 | ASRGL1 protein | | ASRGL1 | | | 11:q12.3 | | | 6.7 | 10 | | | | 8 | | | 19.1 | | AAH64963.1 | | | 32 | | | 4 | | | 32 | | |
| 1300 | ATP synthase beta subunti | | Atp5b | | | 12:q13.3 | | | 5 | 25 | | | | 5 | | | 51.18 | | AAB02288.1 | | | 19 | | | 8 | | | 63 | | |
| 527 | ATP-dependent DNA helicase 2 subunit 1 | | XRCC6 | | | 22:q13.2 | | | 6.7 | 70 | | | | 6 | | | 70.14 | | [CAG47015.1](http://prowl.rockefeller.edu/prowl-cgi/ReadSequence.exe?name=db|1|nr-Homo-sapiens|gi|49457432|emb|CAG47015.1|) | | | 22 | | | 11 | | | 57 | | |
| 1766 | Axin interactor | | AIDA | | | 1:q41 | | | 6.5 | 33 | | | | 6 | | | 35 | | AAH15535.1 | | | 17 | | | 4 | | | 39 | | |
| 1614 | Beta-fibrinogen precursor | | FGB | | | 4:q32.1 | | | 6 | 40 | | | | 6 | | | 38.09 | | AAA52429.1 | | | 46 | | | 24 | | | 89 | | |
| 1461 | Calcyphosine b | | CAPS | | | 19:p13.3 | | | 4.3 | 17 | | | | 5 | | | 17.8 | | NP_542157 | | | 31 | | | 5 | | | 30 | | |
| 1440 | Calcyphosine iso a | | CAPS | | | 19:p13.3 | | | 4.1 | 17 | | | | 5 | | | 21.06 | | NP_004049.1 | | | 57 | | | 12 | | | 50 | | |
| 1654 | Calreticulin precursor | | CALR | | | 19:p13.13 | | | 4.2 | 60 | | | | 4 | | | 48.29 | | NP_004334.1 | | | 15 | | | 4 | | | 33 | | |
| 1496 | Casein kinase 2, alpha prime polypeptide | | CSNK2A2 | | | 16:q21 | | | 4.2 | 16 | | | | 9 | | | 41.37 | | NP_001887.1 | | | 11 | | | 3 | | | 31 | | |
| 1265 | Catechol-O-methyltransferase isoform MB-COMT | | COMT | | | 22:q11.21 | | | 5 | 25 | | | | 5 | | | 30.5 | | NP_000745.1 | | | 28 | | | 4 | | | 46 | | |
| 664 | Chaperonin cont TCP1 | | CCT8 | | | 21:q21.3 | | | 5.7 | 60 | | | | 5 | | | 60.17 | | NP_006576.2 | | | 13 | | | 6 | | | 49 | | |
| 1255 | Chloride intracellular channel 4 | | CLIC4 | | | 1:p36.11 | | | 5.5 | 30 | | | | 6 | | | 28.98 | | NP_039234.1 | | | 26 | | | 5 | | | 34 | | |
| 770 | CSTF 1 | | CSTF1 | | | 20:q13.31 | | | 6.7 | 50 | | | | 6 | | | 49.14 | | NP_001315 | | | 17 | | | 6 | | | 44 | | |
| 1 | 2 | | 3 | | | 4 | | | 5 | 6 | | | | 7 | | | 8 | | 9 | | | 10 | | | 11 | | | 12 | | |
| 1571 | Cyclophilin A, chain A | | PPIA | | | 7:p13 | | | 6.7 | 15 | | | | 8 | | | 18.09 | | 5CYH A | | | 36 | | | 4 | | | 36 | | |
| 1710 | Cyclophilin A, chain A | | PPIA | | | 7:p13 | | | 6.7 | 10 | | | | 8 | | | 18 | | 1BCK A | | | 25 | | | 5 | | | 42 | | |
| 1223 | DDAH2 | | DDAH2 | | | 6:p21.33 | | | 5.7 | 30 | | | | 6 | | | 29.91 | | NP_039268.1 | | | 34 | | | 8 | | | 35 | | |
| 1551 | EEF1 alpha1-like 14 | | EEF1A1 | | | 6:q13 | | | 6.7 | 14 | | | | 9 | | | 43.29 | | AF397403_1 | | | 20 | | | 5 | | | 47 | | |
| 841 | EEF1G | | EEF1G | | | 11:q12.3 | | | 6.5 | 45 | | | | 6 | | | 50.17 | | [AAH07949.2](http://www.ncbi.nlm.nih.gov/entrez/viewer.fcgi?db=protein&val=39644794) | | | 26 | | | 9 | | | 53 | | |
| 1540 | EEF1G protein | | EEF1G | | | 11:q12.3 | | | 6 | 15 | | | | 6 | | | 50.17 | | AAH07949.2 | | | 13 | | | 3 | | | 31 | | |
| 1095 | EIF 1delta | | EEF1D | | | 8:q24.3 | | | 4.5 | 30 | | | | 5 | | | 31.22 | | NP_001951.2 | | | 26 | | | 5 | | | 35 | | |
| 1411 | EIF 4A iso 1 | | EIF4A1 | | | 17:p13.1 | | | 5.5 | 25 | | | | 5 | | | 46.36 | | NP_001407.1 | | | 22 | | | 10 | | | 46 | | |
| 832 | EIF 4A3 | | EIF 4A3 | | | 17:q25.3 | | | 6.7 | 50 | | | | 6 | | | 47.14 | | NP_055555.1 | | | 19 | | | 9 | | | 47 | | |
| 1150 | Emerin | | EMD | | | X:q28 | | | 5 | 30 | | | | 5 | | | 29.03 | | NP_000108.1 | | | 22 | | | 6 | | | 41 | | |
| 1614 | Enolase 1 | | ENO1 | | | 1:p36.23 | | | 6 | 48 | | | | 7 | | | 47.49 | | NP_001419.1 | | | 23 | | | 7 | | | 56 | | |
| 855 | ERP44 | | ERP44 | | | 9:q31.1 | | | 5 | 50 | | | | 5 | | | 47.35 | | NP_055866.1 | | | 11 | | | 3 | | | 43 | | |
| 1416 | GGCT protein | | GGCT | | | 7:p15.1 | | | 4.5 | 18 | | | | 9 | | | 13.1 | | AAH05356.1 | | | 46 | | | 4 | | | 40 | | |
| 1360 | GPD2 protein | | GPD2 | | | 2:q24.1 | | | 5.5 | 25 | | | | 9 | | | 41.71 | | AAH19874.1 | | | 10 | | | 3 | | | 27 | | |
| 1326 | GTP-binding protein Gi alpha-2-chain | GNAI2 | | | 3:p21.31 | | | 6 | | | 22 | | 5 | | | 22.31 | | | AAD12229.1 | | 29 | | | 4 | | | 30 | | |  |
| 1110 | Guanine nucleotide-binding protein, b2 subunit | GNB2 | | | 7:q22.1 | | | 5.5 | | | 30 | | 6 | | | 38.05 | | | NP_005264.2 | | 27 | | | 9 | | | 51 | | |  |
| 524 | Hemopexin precursor | HPX | | | 11:p15.4 | | | 6 | | | 50 | | 7 | | | 52.4 | | | NP_000604.1 | | 21 | | | 7 | | | 67 | | |  |
| 1020 | Hepatoma-derived growth factor iso a | HDGF | | | 1:q23.1 | | | 4.2 | | | 30 | | 5 | | | 26.9 | | | NP_004485.1 | | 45 | | | 10 | | | 32 | | |  |
| 528 | hnRNP 2H9B | HNRNPH3 | | | 10:q21.3 | | | 6.5 | | | 70 | | 7 | | | 31.51 | | | AF132362_1 | | 33 | | | 7 | | | 42 | | |  |
| 972 | hnRNP C | HNRNPC | | | 14:q11.2 | | | 4.5 | | | 35 | | 5 | | | 33.6 | | | AAH07052 | | 32 | | | 7 | | | 45 | | |  |
| 1764 | hnRNP F | HNRNPF | | | 10:q11.21 | | | 5.5 | | | 45 | | 5 | | | 46 | | | NP_004957.1 | | 23 | | | 7 | | | 44 | | |  |
| 708 | hnRNP K | HNRNPK | | | 9:q21.32 | | | 5.5 | | | 50 | | 5 | | | 42.02 | | | [CAI16022.1](http://www.ncbi.nlm.nih.gov/entrez/viewer.fcgi?db=protein&val=55958547) | | 19 | | | 6 | | | 43 | | |  |
| 836 | HSP70 prot 1 | HSPA1A | | | 6:p21.33 | | | 5.5 | | | 50 | | 5 | | | 47.53 | | | [AAA03450.1](http://www.ncbi.nlm.nih.gov/entrez/viewer.fcgi?db=protein&val=414973) | | 22 | | | 7 | | | 62 | | |  |
| 1837 | HSP90,1 alpha iso 2 | HSP90AA1 | | | 14:q32.31 | | | 4.3 | | | 25 | | 5 | | | 85 | | | AAA63194.1 | | 9 | | | 4 | | | 36 | | |  |
| 1439 | HSP90-alpha, chain A | HSP90AA | | | 14:q32.31 | | | 5.5 | | | 18 | | 5 | | | 25.62 | | | 1UY6 A | | 37 | | | 6 | | | 35 | | |  |
| 1313 | HSP90-beta, chain A | HSP90AB1 | | | 6:p21.1 | | | 4.5 | | | 22 | | 5 | | | 24.62 | | | 1UYM A | | 29 | | | 5 | | | 29 | | |  |
| 1706 | Keratin 40kDa | KRT19 | | | 17:q21.2 | | | 4.5 | | | 45 | | 5 | | | 44 | | | AAA36044 | | 32 | | | 12 | | | 88 | | |  |
| 738 | Keratin 7 | KRT7 | | | 12:q13.13 | | | 5.2 | | | 50 | | 5 | | | 51.46 | | | AAH02700.1 | | 27 | | | 10 | | | 66 | | |  |
| 583 | Lamin B2 | LMNB2 | | | 19:p13.3 | | | 5 | | | 70 | | 5 | | | 67.79 | | | NP_116126.2 | | 18 | | | 10 | | | 53 | | |  |
| 584 | Lamin B2 | LMNB2 | | | 19:p13.3 | | | 5.1 | | | 70 | | 5 | | | 67.79 | | | NP_116126.2 | | 21 | | | 13 | | | 75 | | |  |
| 1192 | LAP3/laminin/binding/TUBB | LAP3 | | | 4:p15.32 | | | 5.5 | | | 30 | | 7 | | | 54.77 | | | AAH06199.3 | | 23 | | | 8 | | | 65 | | |  |
| 1614 | Maspin | SERPINB5 | | | 18:q21.33 | | | 6 | | | 45 | | 6 | | | 42.58 | | | AAA18957.1 | | 32 | | | 9 | | | 65 | | |  |
| 1703 | Mithochondrial ATP synthase beta unit | ATP5B | | | 12:q13.3 | | | 4.7 | | | 50 | | 5 | | | 56.54 | | | NP001677 | | 28 | | | 13 | | | 61 | | |  |
| 515 | Moesin | MSN | | | X:q11.1 | | | 6.5 | | | 70 | | 6 | | | 67.9 | | | NP_002435.1 | | 7 | | | 5 | | | 46 | | |  |
| 1019 | mRNA decapping enzyme | DCPS | | | 11:q24.2 | | | 6 | | | 35 | | 6 | | | 38.76 | | | NP_054745.1 | | 12 | | | 5 | | | 43 | | |  |
| 1664 | NADH dehydrogenase ubiquinone fe-S 8 | NDUFS8 | | | 11:q13.2 | | | 5.5 | | | 20 | | 6 | | | 24.2 | | | NP_002487.1 | | 22 | | | 5 | | | 31 | | |  |
| 1487 | NM23A | NME1 | | | 17:q21.33 | | | 6 | | | 16 | | 5 | | | 19.86 | | | NP_937818.1 | | 38 | | | 7 | | | 51 | | |  |
| 1814 | Nuclear chloride channel | CLIC1 | | | 6:p21.33 | | | 5 | | | 27 | | 5 | | | 27.25 | | | AAD26137.1 | | 32 | | | 6 | | | 56 | | |  |
| 1128 | Osteoglycin preprotein | OGN | | | 9:q22.31 | | | 5.7 | | | 30 | | 5 | | | 34.25 | | | NP_054776.1 | | 12 | | | 3 | | | 28 | | |  |
| 1618 | Osteoglycin preprotein | OGN | | | 9:q22.31 | | | 4.7 | | | 30 | | 5 | | | 34.25 | | | NP_054776.1 | | 13 | | | 4 | | | 41 | | |  |
| 1 | 2 | | 3 | | | 4 | | | 5 | 6 | | | | 7 | | | 8 | | 9 | | | 10 | | | 11 | | | 12 | | |
| 1217 | p64 CLCP | CLIC1 | | | 6:p21.33 | | | 5 | | | 17 | | 5 | | | 23.18 | | | CAA61020.1 | | 21 | | | 3 | | | 35 | | |  |
| 1473 | p64 CLCP | CLIC1 | | | 6:p21.33 | | | 5 | | | 17 | | 5 | | | 23.81 | | | CAA61020.1 | | 26 | | | 5 | | | 42 | | |  |
| 1684 | Phosphatase 2A reg sub | PPP2R1A | | | 19:q13.33 | | | 5 | | | 65 | | 5 | | | 66 | | | AAA36399.1 | | 8 | | | 5 | | | 35 | | |  |
| 1327 | Phosphoglycerate mutase 1 | PGAM1 | | | 10:q24.1 | | | 6.5 | | | 25 | | 7 | | | 28.92 | | | AAH62302.1 | | 55 | | | 15 | | | 49 | | |  |
| 1701 | PRDX 6 | PRDX6 | | | 1:q25.1 | | | 6 | | | 15 | | 6 | | | 25.12 | | | NP_004896.1 | | 27 | | | 8 | | | 40 | | |  |
| 1394 | PRDX2 | PRDX2 | | | 19:p13.13 | | | 5.5 | | | 20 | | 6 | | | 21.68 | | | NP_005800.3 | | 62 | | | 10 | | | 40 | | |  |
| 1499 | Proapoptotic caspase adapter protein precursor | PACAP | | | 5:q31.2 | | | 5.2 | | | 15 | | 5 | | | 21.02 | | | AAH21275.1 | | 58 | | | 10 | | | 38 | | |  |
| 1456 | Prostaglandine E synthase 3 | PTGES3 | | | 12:q13.3 | | | 4 | | | 17 | | 4 | | | 18.9 | | | NP_006592 | | 22 | | | 6 | | | 29 | | |  |
| 1720 | Protein disulfid iso | PDIA6 | | | 2:p25.1 | | | 5 | | | 50 | | 5 | | | 46.5 | | | AAB50217 | | 22 | | | 5 | | | 35 | | |  |
| 1012 | Protein disulfide isomerase-rel protein 5 | PDIA5 | | | 3:q21.1 | | | 5.5 | | | 40 | | 5 | | | 46.5 | | | AAB50217.1 | | 22 | | | 5 | | | 34 | | |  |
| 1047 | Protein phosphatase 1 | PPP1CB | | | 2:p23.2 | | | 6 | | | 40 | | 6 | | | 37.95 | | | AAV38549.1 | | 31 | | | 8 | | | 45 | | |  |
| 1174 | Protein phosphatase 1, regulatory subunit 7 | PDILT | | | 16:p12.3 | | | 5.5 | | | 25 | | 5 | | | 36.93 | | | NP_002703.1 | | 22 | | | 7 | | | 36 | | |  |
| 1821 | Protein phosphatase-1 reg sub | PPP1R7 | | | 2:q37.3 | | | 4.5 | | | 45 | | 5 | | | 36.9 | | | AAD26610 | | 22 | | | 6 | | | 27 | | |  |
| 816 | PSMC3 | PSMC3 | | | 11:p11.2 | | | 5 | | | 50 | | 5 | | | 45.52 | | | CAG33012.1 | | 22 | | | 8 | | | 63 | | |  |
| 1604 | Putative c-Myc-resp iso 1 | C6orf108 | | | 6:p21.1 | | | 5 | | | 17 | | 5 | | | 19.2 | | | NP_006434.1 | | 58 | | | 7 | | | 62 | | |  |
| 1612 | Pyrophosphatase 1 | PPA1 | | | 10:q22.1 | | | 5.2 | | | 30 | | 6 | | | 33.1 | | | NP_066952.1 | | 30 | | | 8 | | | 39 | | |  |
| 1843 | RAB1A/abhydrolase | RAB1A | | | 2:p14 | | | 5.2 | | | 20 | | 6 | | | 22.89 | | | NP_004152.1 | | 33 | | | 5 | | | 38 | | |  |
| 1845 | RAB2A | RAB2A | | | 8:q12.1 | | | 6.5 | | | 23 | | 6 | | | 23.7 | | | NP_002856.1 | | 45 | | | 9 | | | 45 | | |  |
| 767 | Rho GTPase act prot 1 | ARHGAP1 | | | 11:p11.2 | | | 6 | | | 50 | | 6 | | | 50.47 | | | [NP_004299.1](http://www.ncbi.nlm.nih.gov/entrez/viewer.fcgi?db=protein&val=4757766) | | 20 | | | 8 | | | 50 | | |  |
| 1163 | Ring finger protein 8 isoform 2 | RNF8 | | | 6:p21.2 | | | 6 | | | 30 | | 6 | | | 51.38 | | | NP_898901.1 | | 13 | | | 5 | | | 28 | | |  |
| 927 | SCC antigen 2 | SERPINB4 | | | 18:q21.33 | | | 5.7 | | | 50 | | 6 | | | 45 | | | AAO92271.1 | | 19 | | | 6 | | | 36 | | |  |
| 1607 | Septin 2 | SEPT 2 | | | 2:q37.3 | | | 6.5 | | | 40 | | 6 | | | 41.7 | | | AAH14455.1 | | 46 | | | 11 | | | 41 | | |  |
| 824 | Serine prot inhibitor | SERPINF1 | | | 17:p13.3 | | | 5.7 | | | 50 | | 6 | | | 46.8 | | | NP_776565 | | 9 | | | 4 | | | 40 | | |  |
| 924 | Serine(cysteine) proteinase inh, clade B | SERPINB1 | | | 6:p25.2 | | | 6.2 | | | 40 | | 6 | | | 42.84 | | | NP_109591.1 | | 28 | | | 10 | | | 38 | | |  |
| 1103 | Splicing factor, Arg/Ser rich 1 iso 1 | SFRS1 | | | 17:q22 | | | 5 | | | 30 | | 11 | | | 27.84 | | | NP_008855.1 | | 33 | | | 8 | | | 58 | | |  |
| 1103 | Splicing factor, Arg/Ser rich 7 | SFRS7 | | | 2:p22.1 | | | 5 | | | 30 | | 12 | | | 27.58 | | | Q16629.1 | | 35 | | | 9 | | | 58 | | |  |
| 1256 | Stratifin | SFN | | | 1:p36.11 | | | 4.2 | | | 30 | | 5 | | | 27.87 | | | NP_006133.1 | | 32 | | | 6 | | | 50 | | |  |
| 923 | Succinate-CoA ligase | SUCLG2 | | | 3:p14.1 | | | 5.5 | | | 45 | | 5 | | | 41.54 | | | AAH47024.1 | | 20 | | | 7 | | | 47 | | |  |
| 1817 | Tropomyosin beta | TPM2 | | | 9:p13.3 | | | 5 | | | 20 | | 5 | | | 29.98 | | | AAF17621.1 | | 25 | | | 10 | | | 60 | | |  |
| 1772 | Tubulin alpha 4A | TUBA4A | | | 19:p13.3 | | | 4.5 | | | 15 | | 5 | | | 50.5 | | | NP_005991 | | 15 | | | 5 | | | 42 | | |  |
| 1820 | Tubulin beta | TUBB | | | 6:p21.33 | | | 4.2 | | | 25 | | 5 | | | 48.15 | | | CAI41893.1 | | 16 | | | 6 | | | 25 | | |  |
| 1197 | Tubulin folding cofactor B | TBCB | | | 19:q13.12 | | | 5 | | | 30 | | 5 | | | 27.66 | | | AAB67716.1 | | 26 | | | 5 | | | 25 | | |  |
| 1032 | Tubulin, alpha | TUBA1B | | | 12:q13.12 | | | 5 | | | 30 | | 5 | | | 50.82 | | | NP_006073.2 | | 32 | | | 12 | | | 70 | | |  |
| 1084 | Tubulin, beta 2B | TUBB2B | | | 6:p25.2 | | | 5.4 | | | 30 | | 5 | | | 50.39 | | | NP_821080.1 | | 38 | | | 12 | | | 66 | | |  |
| 1494 | Transmembrane phosphoinositide 3-phosphatase and tensin homolog 2 | TPTE2 | | | 13:q12.11 | | | 5.5 | | | 16 | | 9 | | | 52.61 | | | CAH73538.1 | | 13 | | | 3 | | | 28 | | |  |
| 1098 | Thioredoxin-like 1 | TXNL1 | | | 18:q21.31 | | | 4.5 | | | 30 | | 5 | | | 32.63 | | | NP_004777.1 | | 42 | | | 8 | | | 51 | | |  |
| 592 | WD repeat-containing protein 1 iso 1 | WDR1 | | | 4:p16.1 | | | 6.7 | | | 70 | | 6 | | | 66.86 | | | NP_059830.1 | | 30 | | | 10 | | | 52 | | |  |
| 708 | Vimentin | VIM | | | 10:p12.33 | | | 5.5 | | | 50 | | 5 | | | 53.62 | | | CAG28618.1 | | 20 | | | 6 | | | 49 | | |  |
| 967 | Vimentin | VIM | | | 10:p12.33 | | | 4.2 | | | 40 | | 5 | | | 53.75 | | | AAA61279.1 | | 28 | | | 14 | | | 45 | | |  |
| 1331 | Vimentin | VIM | | | 10:p12.33 | | | 4.5 | | | 25 | | 5 | | | 35.09 | | | AAA61282.1 | | 45 | | | 12 | | | 55 | | |  |
| 1 | 2 | | 3 | | | 4 | | | 5 | 6 | | | | 7 | | | 8 | | 9 | | | 10 | | | 11 | | | 12 | | |
| 1674 | Vimentin | VIM | | | 10:p12.33 | | | 4.5 | | | 55 | | 5 | | | 53.7 | | | AAA61279.1 | | 40 | | | 19 | | | 57 | | |  |
| 1712 | Vimentin | VIM | | | 10:p12.33 | | | 5.7 | | | 25 | | 5 | | | 53.6 | | | P08670 | | 21 | | | 10 | | | 35 | | |  |
| 1568 | Voltage dependent anion channel 2 | VDAC2 | | | 10:q22.2 | | | 6 | | | 15 | | 8 | | | 30 | | | CAH73106 | | 30 | | | 5 | | | 30 | | |  |
| 1470 | 14-3-3 protein | YWHAE | | | 7:p13.3 | | | 4.5 | | | 17 | | 5 | | | 29.33 | | | AAC37659.1 | | 22 | | | 5 | | | 33 | | |  |
| 1427 | ZNF510 protein | ZNF510 | | | 9:q22.33 | | | 4.2 | | | 18 | | 10 | | | 75.46 | | | AAH68587.1 | | 7 | | | 4 | | | 37 | | |  |
| 1435 | ZNF510 protein | ZNF510 | | | 9:q22.33 | | | 5.5 | | | 18 | | 10 | | | 75.46 | | | AAH68587.1 | | 7 | | | 4 | | | 36 | | |  |
|  |  |  | | |  | | |  | | |  | |  | | |  | | |  | |  | | |  | | |  | | |  |
|  |  |  | | |  | | |  | | |  | |  | | |  | | |  | |  | | |  | | |  | | |  |
| Note: | |  | |  | | |  | | |  | |  | | |  | | |  | |  | | |  | | |  | | |  | |
| 1 - number of a protein spot on 2-D gel, | | | |  | | |  | | |  | |  | | |  | | |  | |  | | |  | | |  | | |  | |
| 2 - protein name, | |  | |  | | |  | | |  | |  | | |  | | |  | |  | | |  | | |  | | |  | |
| 3 - gene ontology name, | |  | |  | | |  | | |  | |  | | |  | | |  | |  | | |  | | |  | | |  | |
| 4 - gene location on chromosomes, | |  | |  | | |  | | |  | |  | | |  | | |  | |  | | |  | | |  | | |  | |
| 5 - isoelectric point of a protein spots according to the position on 2-D gel, | | | | | | | | | | | | | | | | | |  | |  | | |  | | |  | | |  | |
| 6 - protein mass of a protein according to the position of a spot on 2-D gel, | | | | | | | | | | | | | | | | | |  | |  | | |  | | |  | | |  | |
| 7 - isoelectric point of a protein spot as provided by ProFound search database, | | | | | | | | | | | | | | | | | | | | | | | | | | | | |  | |
| 8 - protein mass as provided by ProFound search database, | | | | | | | | | | | | | | | | | | | | | | | | | |  | | |  | |
| 9 - protein accession number according to NCBI, | | | |  | | |  | | |  | |  | | |  | | |  | |  | | |  | | |  | | |  | |
| 10 - matching of the experimental peptide sequence to a peptide sequence provided by NCBI, | | | | | | | | | | | | | | | | | | | | | | | | | | | | |  | |
| 11 - number of identified peptides that matched to the peptides of a peptide sequence provided by NCBI, | | | | | | | | | | | | | | | | | | | | | | | | | | | | |  | |
| 12 - total amount of peptides in a peptide sequence provided by NCBI. | | | | | | | | | | | | | | | | | | | | | | | | | | | | |  | |
