# Supplementary material for: Impact of genomic stability on protein expression in endometrioid endometrial cancer
Source: Br J Cancer. 2012 Mar 13;106(7):1297–305. doi: 10.1038/bjc.2012.67 (PMC3314786; doi:10.1038/bjc.2012.67)
Supplement: Supplementary Table 2 [file bjc201267x2.doc]

Supplementary table 2. Expression of identified proteins at various stages of EEC and SCC:

| 1. proteins over-expressed in EEC, stage I (FIGO, 1998) | | | | | | | | | | |  |  |
| --- | --- | --- | --- | --- | --- | --- | --- | --- | --- | --- | --- | --- |
| Protein spot № | GO | genomically stable EEC, stage 1B compared to stage 1A | |  | Protein spot № | GO | | genomically unstable EEC, 1C compared to stage 1B | |  |  |  |
|  |  | fold |  |  |  |  |  | fold |  |  |  |  |
| 1 | 2 | 3 |  |  | 1 | 2 | | 3 |  |  |  |  |
| 1837 | HSP90AA1 | *1.3634* |  |  | 1456 | PTGES3 | | 1.54514 |  |  |  |  |
|  |  |  |  |  | 1300 | Atp5b | | 2.14762 |  |  |  |  |
|  |  |  |  |  |  |  | |  |  |  |  |  |
| 1. proteins over- and under-expressed at various stages of SCC, (FIGO, 1994) | | | | | | | | | | | | |
| Protein spot № | GO | stage 1B2 compared to stage 1B1 | stage 2A compared to stage 1B1 | stage 2A compared to stage 1B2 | stage III compared to stage 1B1 | stage III compared to stage 1B2 | stage III compared to stage 2A | |  |  |  |  |
|  |  | fold | fold | fold | fold | fold | fold | |  |  |  |  |
| 1 | 2 | 3 | | | | | | |  |  |  |  |
| 1766 | AIDA | 1.5439 | 1.6079 | 1.04146 | 1.7043 | 1.103881 | | 1.05994 |  |  |  |  |
| 528 | ANXA1 | 1.8764 | 1.6263 | 0.8667 | 2.4306 | 1.295351 | | 1.49457 |  |  |  |  |
| 1378 | APOA1 | 0.2777 | 0.5751 | 2.07106 | 0.6718 | 2.41947 | | 1.16823 |  |  |  |  |
| 1473 | CLIC1 | 0.4205 | 0.9115 | 2.16756 | 0.5429 | 1.290979 | | 0.59559 |  |  |  |  |
| 1604 | C6orf108 | 0.6348 | 0.8361 | 1.31714 | 1.1581 | 1.824368 | | 1.3851 |  |  |  |  |
| 1265 | COMT | *0.7083* | 0.8275 | 1.16834 | 0.829 | 1.17046 | | 1.00181 |  |  |  |  |
| 1019 | DCPS | 0.5212 | 0.7098 | 1.36172 | 1.0334 | 1.982523 | | *1.4559* |  |  |  |  |
| 1551 | EEF1A1 | 0.4661 | 0.8318 | 1.78462 | 0.9364 | 2.009152 | | 1.12582 |  |  |  |  |
| 1540 | EEF1G | 0.6971 | 1.0938 | 1.56896 | 0.9242 | 1.325754 | | 0.84499 |  |  |  |  |
| 832 | EIF 4A3 | 1.6228 | 1.0142 | 0.62497 | 1.7781 | 1.09574 | | 1.75327 |  |  |  |  |
| 1411 | EIF4A1 | 0.3788 | 0.6657 | 1.75756 | 0.3744 | 0.988364 | | 0.56235 |  |  |  |  |
| 1150 | EMD | 1.4468 | 2.3326 | 1.61223 | 2.8646 | 1.979933 | | 1.22807 |  |  |  |  |
| 1416 | GGCT | 0.4798 | 0.8008 | 1.66889 | 0.7995 | 1.666351 | | 0.99848 |  |  |  |  |
| 1360 | GPD2 | 0.6342 | 0.9583 | 1.51092 | 0.7844 | 1.236861 | | 0.81861 |  |  |  |  |
| 528 | HNRNPH3 | 1.8764 | 1.6263 | 0.8667 | 2.4306 | 1.295351 | | 1.49457 |  |  |  |  |
| 1192 | LAP3 | 0.7005 | 1.3498 | 1.92701 | 1.4669 | 2.094142 | | 1.08673 |  |  |  |  |
| 1664 | NDUFS8 | 0.8078 | 0.802 | 0.99287 | 1.2041 | 1.490549 | | 1.50125 |  |  |  |  |
| 1128 | OGN | 0.5664 | 1.0709 | 1.89074 | 0.9356 | 1.651849 | | 0.87365 |  |  |  |  |
| 1618 | OGN | 0.4992 | 0.924 | 1.85095 | 0.6678 | 1.337726 | | 0.72272 |  |  |  |  |
| 1257 | PGLS | 1.4688 | 1.3306 | 0.90593 | 1.9202 | 1.307375 | | 1.44313 |  |  |  |  |
| 1612 | PPA1 | *0.7727* | 1.0901 | 1.41071 | *1.2027* | 1.556419 | | 1.10329 |  |  |  |  |
| 1571 | PPIA | 1.0868 | 1.5093 | 1.3888 | 0.7123 | 0.65548 | | 0.47198 |  |  |  |  |
| 1456 | PTGES3 | 0.4687 | 0.6779 | 1.44613 | 0.4412 | 0.941148 | | 0.6508 |  |  |  |  |
| 1163 | RNF8 | 1.5689 | 1.403 | 0.89429 | 3.5798 | 2.281751 | | 2.55146 |  |  |  |  |
| 1494 | TPTE2 | 0.4389 | 0.8582 | 1.9552 | 0.6493 | 1.479184 | | 0.75654 |  |  |  |  |
| 1084 | TUBB2B | 0.8584 | 1.5019 | 1.74955 | 1.0989 | 1.280129 | | 0.73169 |  |  |  |  |
| 1568 | VDAC2 | 0.6009 | 1.6941 | 2.81914 | *0.8457* | 1.407314 | | 0.4992 |  |  |  |  |
| 1712 | VIM | 0.6881 | 0.2872 | 0.41742 | 1.0617 | 1.542915 | | 3.69629 |  |  |  |  |
| 1470 | YWHAE | 0.6258 | 1.4887 | 2.37887 | 0.9135 | 1.459753 | | 0.61363 |  |  |  |  |
| Note: | |  |  |  |  |  | |  |  |  |  |  |
| 1 - number of a protein spot on 2-D gel, | | | | | | | |  |  |  |  |  |
| 2 - gene ontology name, | | | | |  |  | |  |  |  |  |  |
| 3 - ratio between the expression of a protein spot at various stages of EEC and SCC: | | | | | | | | | | | | |
|  | - fold changes <1.5 are presented in Italic script, | | | | | | | | |  |  |  |
|  | - highlighted numbers correspond to statistically significant changes where green | | | | | | | | | | | |
|  | corresponds to under-expression and red to over-expression (cut-off 1.5, p<0.05) | | | | | | | | | | | |
