# Supplementary material for: Impact of genomic stability on protein expression in endometrioid endometrial cancer
Source: Br J Cancer. 2012 Mar 13;106(7):1297–305. doi: 10.1038/bjc.2012.67 (PMC3314786; doi:10.1038/bjc.2012.67)
Supplement: Supplementary Table 3 [file bjc201267x3.doc]

Supplementary table 3: Distribution of the identified proteins according to chromosomal gains/losses and with the analysis of clinical relevance - analysis of the published chromosomal genomic hybridization (CGH) studies of EEC and SCC.

| **Chromosomes** | **Gains/losses** | **Clustering of proteins by up- and down-regulation and location of corresponding genes** | | | | | | | | **Chromosomal changes during the development of EEC and SCC and clinical relevance** | | | | | | | | | | | | | |
| --- | --- | --- | --- | --- | --- | --- | --- | --- | --- | --- | --- | --- | --- | --- | --- | --- | --- | --- | --- | --- | --- | --- | --- |
|  |  | **Over-expressed in:** | |  | **Under-expressed in cancer:** | | | | | **Changes during carcinogenesis leading to:** | | |  |  | **Prognosis for EEC patients and related parameters** | | | | | | |  |  |
|  |  | **Genomically unstable EEC and SCC** | | | **Genomically unstable and stable EEC**  **but up-regulated in SCC** | | **Genomically unstable EEC and SCC** | **SCC** | **Genomically unstable and stable EEC and SCC** | **EEC** | |  | **SCC (FIGO, 1994)** | |  | |  | **Grade** | | **Stage (FIGO, 1988)** | | | |
|  |  |  |  |  |  |  |  |  |  | **AH**  (Baloglu *et al*, 2001) | **CH →AH →EEC** | **EEC** | **CIN3→IA**  (Heselmeyer *et al,* 1996) | **IIB-IV**  (Heselmeyer *et al,* 1997) | **Worse sur-**  **vival** | **High metasta-tic poten-tial** | | **G3** | **G2** | **I** | **II** | **III** | **IV** |
| a | b | c | | | d | | e | f | g | h | i | j | k | l | m | n | | o | p | q | r | s | t |
| **1p** | **↑** | Gu: 1:p36.11 (SFN, #1256) |  |  | SCC: 1:p36.11 (CLIC4, #1255) |  |  |  |  | ↑ of 1 |  | ↑ at 1p13.2, 1p36.2-36.1 (Schulten, *et al*, 2004) |  | ↑ at 1p |  |  | |  |  |  |  |  |  |
|  | **↓** |  |  |  | Gu,Gs: 1:p36.11 (CLIC4, #1255) | 1:p36.23 (ENO1, #1614) |  |  |  |  | ↓ at 1p: 1pter-p36 (Kiechle *et al*, 2000) |  |  |  |  |  | |  |  |  |  |  |  |
| **1q** | **↑** | Gu,SCC: 1:q25.1 (PRDX6, #1701) |  |  | SCC: 1:q24.1 (ALDH9A1, #1717) |  | Gs: 1:q41 (AIDA, #1766) |  |  | ↑ of 1 | ↑ in 1q (Suzuki *et al*, 1997) | ↑ in 1q (Suzuki *et al*, 1997, Schulten *et al*, 2004) |  | ↑ at 1q | ↑ at 1q25-42 (Levan, et al, 2006) | ↑ at 1q25-42 (Levan, et al, 2006) | | ↑ at 1q25-42 (Levan, et al, 2006), ↑ of 1 (Baloglu *et al*, 2001) |  | ↑ at 1q25-42, more invasive, bad prognosis (Levan et al, 2006) | ↑ at 1q25-42, more invasive (Levan, et al, 2006) | ↑ at 1q25-42 (Levan, et al, 2006) | ↑ at 1q25-42 (Levan et al, 2006) |
| a | b | c | | | d | | e | f | g | h | i | j | k | l | m | n | | o | p | q | r | s | t |
| **1q** | **↓** |  |  |  | Gu: 1:q24.1 (ALDH9A1, #1717) |  | Gu: 1:q41 (AIDA, #1766) | 1:q23.1 (HDGF, #1020) |  |  |  |  |  |  |  |  | |  |  |  |  |  |  |
| **2p** | **↑** | Gu,SCC: 2:p14 (ANXA4, #1757) | Gu: 2:p14 (RAB1A, #1843) |  |  |  |  |  |  |  |  | ↑ in 2p (Suzuki *et al*, 1997) |  | ↑ at 2p |  |  | |  |  |  |  |  |  |
|  |  | Gu, SCC: 2:p25.3 (ACP1, #1527) |  |  |  |  |  |  |  |  |  |  |  |  |  |  | |  |  |  |  |  |  |
| **2p** | **↓** |  |  |  |  |  |  | 2:p23.2 (PPP1CB, #1047) | 2:p22.1 (SFRS7, #1103) |  |  | ↓ at 2 (Baloglu *et al*, 2001) |  | ↓ at 2pter-24 |  |  | |  |  |  |  |  |  |
| **2q** | **↑** | Gu: 2:q24.1 (GPD2, #1360) |  |  |  |  |  |  |  |  |  | ↑ in 2q (Suzuki *et al*, 1997) |  |  |  |  | |  |  |  |  |  |  |
|  | **↓** |  |  |  |  | 2:q37.3 (PPP1R7, #1821) |  | 2:q37.3 (SEPT2, #1607) |  |  |  |  |  | ↓ at 2q |  |  | |  |  |  |  |  |  |
| **3p** | **↑** | Gu: 3:p21.31 (GNAI2, #1326) |  |  |  |  |  |  |  |  |  | microsomally unstable - ↑ in 3p (Suzuki *et al*, 1997) |  |  |  |  | |  |  |  |  |  |  |
|  | **↓** |  |  |  |  |  | 3:p14.1 (SULG2, #923) |  |  |  |  | microsomally unstable - ↓ in 3p (Suzuki, *et al*, 1997), ↓ at 3p12.1-12.2, 3p14.1-14.2, 3p21.32, 3p24.2-26.2 (Baloglu *et al*, 2001) | from CIN2: ↓ at 3p | ↓ at 3p |  |  | |  |  |  |  |  |  |
| a | b | c | | | d | | e | f | g | h | i | j | k | l | m | n | | o | p | q | r | s | t |
| **3q** | **↑** | Gu: 3:q21.1 (PDIA5, #1012) |  |  |  |  |  |  |  |  |  | ↑ in 3q in microsomally stable and unstable (Suzuki *et al*, 1997), ↑ at 3q26.3 (Schulten *et al*, 2004) | from CIN2: ↑ at 3q, esp 3q24-28 | ↑ at 3q |  | ↑ at 3q26.1-qter (Levan et al, 2006) | |  |  |  | Bad prognosis at st. II: ↑ at 3q26.1-qter (Levan et al, 2006) |  |  |
|  | **↓** |  |  |  |  |  |  |  |  |  |  | ↓ at 3q21.3-28 (Baloglu *et al*, 2001) |  |  |  |  | |  |  |  |  |  |  |
| **4p** | **↑** |  |  | Gu,SCC: 4:p15.32 (LAP3, #1192) |  |  |  | SCC: 4:p16.1 (WDR1, #592) |  |  |  |  |  |  |  |  | |  |  |  |  |  |  |
|  | **↓** |  |  |  |  |  |  |  |  |  |  |  |  | ↓ at 4p |  |  | |  |  |  |  |  | ↓ at 4p15.2-p12 (Levan et al, 2006) |
| **4q** | **↑** | Gu: 4:q13.3 (ALB, #935, #1410) |  | Gu,SCC: 4:q27 (ANXA5,N-, #1560) |  |  |  |  |  |  | ↑ at 4q (Kiechle *et al*, 2000) |  |  |  |  |  | |  |  |  |  |  |  |
| **4q** | **↓** |  |  |  |  | 4:q32.1 (FGG, #792,#1614) |  |  | 4:q27 (ANXA5,full, #1724) |  |  | ↓ in 4q in microsomally stable and unstable (Suzuki *et al*, 1997) | from CIN3: ↓ at 4q | ↓ at 4q |  | ↓ at 4q22-qter (Levan et al, 2006) | |  |  | ↓ at 4q22-qter (Levan et al, 2006) |  |  | ↓ at 4q22-qter (Levan et al, 2006) |
| a | b | c | | | d | | e | f | g | h | i | j | k | l | m | n | | o | p | q | r | s | t |
| **5q** | **↑** |  |  | Gu,SCC: 5:q31.2 (PACAP, #1499) |  |  |  |  |  |  |  | ↑ in 5q in microsomally stable and unstable (Suzuki *et al*, 1997) |  |  |  |  | |  |  |  |  |  |  |
|  | **↓** |  |  |  |  |  |  |  |  |  |  | ↓ in 5q in microsomally stable and unstable (Suzuki *et al*, 1997) |  |  |  |  | |  |  |  |  |  |  |
| **6p** | **↑** | Gu: 6:p21.33 (TUBB, #1820) | Gu: 6:p21.33 (CLIC1, #1473,#1814) | Gu,SCC: 6:p21.1 (HSP90AB1, #1313) | SCC: 6:p25.2 (TUBB2b, #1084) | SCC: 6:p21.33 (HSPA1A, #836) |  |  |  |  |  | ↑ at 6p in microsomally stable and unstable (Suzuki *et al*, 1997) | ↑ at 6p | ↑ at 6p |  |  | |  |  |  |  | ↑ at 6pter-p22, ↑ at 6pter-q23(Sonoda *et al*, 1997) |  |
|  | **↓** | SCC: 6:p21.33 (TUBB, #1820) | Gs,SCC: 6:p21.33 (CLIC1, #1473,#1814) |  |  | Gu,Gs: 6:p21.33 (HSPA1A, #836) |  | 6:p21.1 (C6orf108, #1604)  6:p21.33 (DDAH2, #1223)  6:p21.2 (RNF8, #1163) |  |  |  |  |  | ↓ at 6p |  |  | |  |  |  |  |  |  |
| **6q** | **↑** | Gu: 6:q13 (EEF1A1, #1551) |  |  |  |  |  |  |  |  |  | ↑ at 6q in microsomally stable and unstable (Suzuki *et al*, 1997) |  |  |  |  | |  |  |  |  | ↑ at 6pter-q23, ↑ at 6q (Levan et al, 2006) |  |
|  | **↓** |  |  |  |  |  |  |  |  |  |  |  | ↓ at 6q | ↓ at 6q |  |  | |  |  |  |  |  |  |
| a | b | c | | | d | | e | f | g | h | i | j | k | l | m | n | | o | p | q | r | s | t |
| **7p** | **↑** | 7:p13.3 (YWHAE, #1470)  Gu,Gs: 7:p15.1 (GGCT, #1416) | Gu,SCC: 7:p13 (PPIA, #1710) | Gu,SCC: 7:p13 (PPIA, #1571)  Gu, SCC: 7:p22.1 (ACTB, #1562, #1205) | Gu, SCC: 7:p22.1 (ACTB, #1196, #1171, #1189) |  |  |  |  | ↑ of 7 |  | ↑ of chr 7 (Schulten *et al*, 2004, (Sonoda *et al*, 1997), ↑ at 7p (Suzuki *et al*, 1997) | ↑ at 7p | ↑ at 7p |  |  | |  |  |  |  |  |  |
|  | **↓** | SCC: 7:p15.1 (GGCT, #1416) |  |  |  |  |  |  |  |  |  |  |  |  |  |  | |  |  |  |  |  |  |
| **7q** | **↑** |  |  |  |  |  |  |  |  | ↑ of 7 |  | ↑ at 7q31 (Sonoda*et al*, 1997) | ↑ at 7q | ↑ at 7q |  |  | |  |  |  |  | ↑ at 7q11.2-q21 (Levan et al, 2006), ↑ at 7q21-q33 (Sonoda *et al*, 1997) |  |
|  | **↓** |  |  |  |  |  |  |  | 7:q22.1 (GNB2, #1110)  7:q11.23 (HSPB1, #1817) |  |  | ↓ at 7q (Suzuki *et al*, 1997) |  | ↓ at 7q |  |  | |  |  |  |  |  |  |
| **8q** | **↑** | Gu: 8:q12.1 (RAB2A, #1845) |  |  |  |  |  |  |  | ↑ at 8q12-13 | ↑ in 8q (Suzuki *et al*, 1997, (Sonoda *et al*, 1997) | ↑ at 8q (Suzuki *et al*, 1997),  25, (Sonoda *et al*, 1997) |  | ↑ at 8q |  | ↑ at 8q21-22 (Levan, et al, 2006) | | ↑ at 8q21-22, 8q22-qter (Levan, et al, 2006), loses at 8q11.2, 8q21.1-21.2, 8q 24.1-24.2 |  | Bad prognosis at st.I: ↑ at 8q21-22, 8q22-qter (Levan et al, 2006) | ↑ at 8q21-22, 8q22-qter (Levan et al, 2006) |  | ↑ at 8q21-22, 8q22-qter (Levan et al, 2006) |
| a | b | c | | | d | | e | f | g | h | i | j | k | l | m | n | | o | p | q | r | s | t |
| **8q** | **↓** |  |  |  |  |  |  |  | 8:q24.3 (EEF1D, #1095) | ↓ at 8q13-24.2 |  |  |  |  |  |  | |  |  |  |  |  |  |
| **9q** | **↑** | Gu,SCC: 9:q22.33 (ZNF510, #1427) |  | Gu,SCC: 9:q22.33 (ZNF510, #1435) |  | SCC: 9:q21.32 (HNRNPK, #708) | Gs: 9:q22.31 (OGN, #1128, #1618) |  |  |  |  |  | ↑ in 9q | ↑ in 9q |  |  | |  |  |  |  |  |  |
|  | **↓** |  |  |  |  | 9:q31.1 (ERP44, #855)  Gu,Gs: 9:q21.32 (HNRNPK, #708) | Gu,SCC: 9:q22.31 (OGN, #1128, #1618) |  | 9:q21.13 (ANXA1, #528) | ↓ at 9q13-22, 9q34 |  | ↓ at 9q |  |  |  | ↓ at 9q33-qter (Levan et al, 2006) | | ↓ of 9 (Baloglu *et al*, 2001) |  |  | Bad prognosis at st. II: ↓ at 9q33-qter (Levan et al, 2006) |  |  |
| **10p** | **↑** |  |  | 10:p12.33 (VIM, #1331) |  | 10:p12.33 (VIM, #1712 #708) |  |  |  |  | ↑ at 10 (Sonoda *et al*, 1997), ↑ at 10p (Suzuki *et al*, 1997) | ↑ at 10 (Schulten, *et al*, 2004, Sonoda *et al*, 1997), ↑ at 10p (Suzuki *et al*, 1997) |  | ↓/↑ in 10p |  |  | |  | ↑ at 10p (Levan et al, 2006) | ↑ at 10 (Sonoda*et al*, 1997) | ↑ at 10p (Levan et al, 2006) |  | ↑ at 10p (Levan et al, 2006) |
| **10p** | **↓** |  |  |  |  |  |  |  | 10:p12.33 (VIM, #967,1674) | ↓ at 10p13-14 |  |  | ↓ in 10p | ↓/↑ in 10p |  |  | |  |  |  |  |  |  |
| **10q** | **↑** | Gu: 10:q22.2 (VDAC2, #1568)  Gu: 10:q22.2 (ADK, #849) | Gu: 10:q24.1 (PGAM1, #1327) |  | SCC: 10:q11.21 (HNRNPF, #1764) |  |  |  |  | ↑ at 10q21.1 -21.2 |  | ↑ and ↓ at 10q (Suzuki *et al*, 1997) |  | ↓/↑ in 10q |  |  | |  | ↑ at 10q21-q23 (Levan et al, 2006) |  | ↑ at 10q21-q23 (Levan et al, 2006) |  | ↑ at 10q21-q23 (Levan et al, 2006) |
|  | **↓** |  |  |  | Gu,Gs: 10:q11.21 (HNRNPF, #1764) |  |  | 10:q22.1 (PPA1, #1612) | 10:q21.3 (HNRNPH3, #528) | ↓ at 10q11.2, q21.3-23.2, q24.1-26.2 |  | ↑ and ↓ at 10q (Suzuki *et al*, 1997) | ↓ in 10q | ↓/↑ in 10q |  |  | |  |  |  |  |  |  |
| **11p** | **↑** |  |  |  | SCC: 11:p15.4 (HPX, #524) |  |  |  |  |  |  | ↑ at 11p15-p11.2 (Sonoda*et al*, 1997) |  |  |  |  | |  |  |  |  |  |  |
| a | b | c | | | d | | e | f | g | h | i | j | k | l | m | n | | o | p | q | r | s | t |
| **11p** | **↓** |  |  |  | Gu,Gs: 11:p15.4 (HPX, #524) | 11:p11.2 (PSMC3, #816)  11:p11.2 (ARHGAP1, #767) |  | 11:p15.5 (TALDO1, #1043) |  |  |  | ↓ at 11p in microsomally ustable (Suzuki *et al*, 1997) | ↓ at 11p | ↓ at 11p |  |  | |  |  |  |  |  |  |
| **11q** | **↑** | Gu,SCC: 11:q12.3 (ASRGL1, #1603) |  | Gu, SCC: 11:q12.3 (EEF1G, #1540) |  |  |  |  |  |  |  | ↑ at 11q22-23, 11q14-qter (Sonoda*et al*, 1997), ↑ at 11q |  |  |  |  | |  |  |  |  |  |  |
|  | **↓** |  |  |  |  |  | 11:q12.3 (EEF1G, #841) | 11:q24.2 (DCPS, #1019) |  |  |  | ↓ at 11q in microsomally unstable (Suzuki *et al*, 1997) | ↓ at 11q | ↓ at 11q | ↓ at 11q22-qter (Levan et al, 2006) |  | |  |  |  |  |  |  |
| **12q** | **↑** | Gu: 12:q13.3 (PTGES3, #1456) | Gu: 12:q13.3 (ATP5B, #1300) |  | SCC: 12:q13.12 (TUBA1B, #708,#1032)  SCC: 12:q13.3 (ATP5B, #1703) | SCC: 12:q13.13 (KRT7, #738) |  |  |  |  |  | ↑ at 12pter-12q22 (Sonoda*et al*, 1997) |  | ↑ at 12q |  |  | |  |  |  | Bad prognosis at st. II: ↑ at 12q12-q23 (Levan et al, 2006 |  |  |
|  | **↓** | Gs, SCC: 12:q13.3 (PTGES3, #1456) | Gs,SCC: 12:q13.3 (ATP5B, #1300) |  | Gu,Gs: 12:q13.3 (ATP5B, #1703) | Gu,Gs: 12:q13.13 (KRT7, #738) |  |  |  |  |  | ↓ at 12q in microsomally unstable (Suzuki *et al*, 1997) |  |  |  |  | |  |  |  |  |  |  |
| **13q** | **↑** | Gu: 13:q12.11 (TPTE2, #1494) |  |  |  |  |  |  |  |  |  | ↑ at 13q21-qter (Sonoda *et al*, 1997) |  |  |  |  | | ↑ at 13q21-qter (Suzuki *et al*, 1997) |  |  |  | ↑ at 13q21-qter (Sonoda *et al*, 1997) |  |
|  | **↓** | Gs,SCC: 13:q12.11 (TPTE2, #1494) |  |  |  |  |  |  |  |  |  | ↓ at 13q in microsomally unstable (Suzuki *et al*, 1997) | ↓ at 13q | ↓ at 13q | ↓ at 13q12-q14-q32 (Levan et al, 2006) |  | |  |  |  | ↓ at 13q12-q14-q32 (Levan et al, 2006) |  | ↓ at 13q14-q32 (Levan et al,2006) |
| a | b | c | | | d | | e | f | g | h | i | j | k | l | m | n | | o | p | q | r | s | t |
| **14q** | **↑** | Gu,SCC: 14:q32.31 (HSP90AA, #1439, #1837) |  |  |  |  |  |  |  |  |  | ↑/↓ at 14q |  | ↑/↓ at 14q |  |  | |  |  |  |  |  |  |
| **14q** | **↓** |  |  |  |  | 14:q32.13 (SERPINA1, #659) |  | 14:q11.2 (HNRNPC, #972) | 14:q32.12 (FBLN5, #676) |  |  | ↑/↓ at 14q, ↓ at 14q in microsomally unstable (Suzuki *et al*, 1997) |  | ↑/↓ at 14q |  |  | |  |  |  |  |  |  |
| **16p** | **↑** | Gu: 16:p12.3 (PDILT, #1174) |  |  | SCC: 16:p13.3 (DCI, #1258) |  |  |  |  |  | ↑ at 16p12 (Kiechle *et al*, 2000) |  |  | ↑ at 16p |  |  | |  |  |  |  |  |  |
|  | **↓** | SCC: 16:p12.3 (PDILT, #1174) |  |  | Gu: 16:p13.3 (DCI, #1258) |  |  |  |  |  | ↓ at 16p13 (Kiechle *et al*, 2000) |  |  |  |  |  | |  |  |  |  |  |  |
| **16q** | **↑** |  |  | Gu,SCC: 16:q21 (CSNK2A2, #1496) |  |  |  |  |  |  |  |  |  | ↑/↓ at 16q |  |  | |  |  |  |  |  |  |
|  | **↓** |  |  |  |  |  |  |  |  |  |  | ↓ at 16q |  | ↑/↓ at 16q | ↓ at 16q21-qter (Levan et al, 2006) | ↓ at 16q21-qter (Levan et al, 2006) | |  |  | Bad prognosis at st. I: ↓ at 16q21-qter (Levan et al, 2006) |  |  |  |
| **17p** | **↑** | Gu: 17:p13.1 (EIF4A1, #1411) |  |  |  |  |  |  |  |  |  |  | ↑ at 17p | ↑/↓ at 17p |  |  | |  |  |  |  |  |  |
|  | **↓** | SCC: 17:p13.1 (EIF4A1, #1411) |  |  |  |  |  |  | 17:p13.3 (SERPINF1, #824) |  |  | ↓ at 17p (Suzuki *et al*, 1997) |  | ↑/↓ at 17p |  |  | |  |  |  | Bad prognosis at st. II: ↓ at 17p (Levan et al, 2006) |  |  |
| **17q** | **↑** |  | Gu,SCC: 17:q25.3 (ACTG, #1268) | Gu,SCC: 17:q25.3 (ACTG1, #1529)  Gu,SCC: 17:q21.33 (NME1, #1487) |  |  |  |  |  |  |  | ↑ at 17q (Suzuki *et al*, 1997), 17q23 (Schulten *et al*, 2004) | ↑ at 17q | ↑ at 17q |  |  | |  |  |  |  |  |  |
| a | b | c | | | d | | e | f | g | h | i | j | k | l | m | n | | o | p | q | r | s | t |
| **17q** | **↓** |  |  |  |  | 17:q25.3 (EIF4A3, #832) |  | 17:q22 (SFRS1, #1103) | ↓ at 17q12-21.1, q22, q25 |  |  |  |  |  |  |  | |  | ↓ at 17q22-qter (Levan et al, 2006) |  |  |  |  |
| **18q** | **↑** |  |  |  | 18:q21.33 (SERPINB4, #927) | 18:q21.33 (SERPINB5, #1614) |  |  |  |  |  | ↑ at 18q in microsomally unstable (Suzuki *et al*, 1997) |  | ↓/↑ at 18q |  |  | |  |  |  |  |  |  |
|  | **↓** |  |  |  | 18:q21.33 (SERPINB4, #927) | 18:q21.33 (SERPINB5, #1614) |  | 18:q21.31 (TXNL1, #1098) |  | ↓ at 18q |  |  |  | ↓/↑ at 18q | ↓ at 18q21-qter (Levan et al, 2006) |  | |  |  |  | Bad prognosis at st.II: ↓ at 18q21-qter (Levan et al, 2006) |  |  |
| **19p** | **↑** | 19:p13.3  (CAPSb, #1461) | SCC: 19:p13.13 (PRDX2, #1394) |  | SCC: 19:p13.3  (LMNB2, #583,#584) |  |  |  |  |  |  | ↑ at 19p in microsomally unstable (Suzuki *et al*, 1997) | ↑ at 19p | ↑ at 19p | ↑ at 19pter-p13.1 (Levan et al, 2006) |  | |  |  | ↑ at 19pter-p13.1 in more invasive (Levan et al, 2006) |  | ↑ at 19pter-p13.1 (Levan et al, 2006) |  |
| **19p** | **↑** | Gu,SCC: 19:p13.3 (TUBA4A, #1772) | Gu: 19:p13.3  (CAPSa, #1440) |  |  |  |  |  |  |  |  |  |  |  |  |  | |  |  |  |  |  |  |
|  | **↓** |  | SCC: 19:p13.3  (CAPSa, #1440) |  | Gu,Gs: 19:p13.3  (LMNB2, #583,#584) | 19:p13.11 (PGLS, #1257) |  |  |  |  |  |  |  |  |  |  | |  |  |  |  |  |  |
| a | b | c | | | d | | e | f | g | h | i | j | k | l | m | n | | o | p | q | r | s | t |
| **19q** | **↑** |  |  |  |  | SCC: 19:q13.33 (PPP2R1A, #1684) |  |  |  |  |  | ↑ in 19q (Suzuki *et al*, 1997) | ↑ at 19q | ↑ at 19q | at stage II - ↑ at 19q13.1-13.3 (Levan et al, 2006) |  | |  |  | ↑ at 19q13.1-13.3 in more invasive (Levan et al, 2006) | Bad prognosis at st. II: increased copies at 19q13.1-q13.3 (Levan et al, 2006) | ↑ at 19q13.1-13.3 (Levan et al, 2006) |  |
|  | **↓** |  |  |  |  | Gu,Gs: 19:q13.33 (PPP2R1A, #1684) |  | SCC: 19:q13.12 (TBCB, #1197) |  |  |  |  |  |  |  |  | |  |  |  |  |  |  |
| **20q** | **↑** |  |  |  |  |  |  |  |  |  |  | ↑ at 20q in microsomally unstable (Suzuki *et al*, 1997) | ↑ at 20q | ↑ at 20q | ↑ at 20q11.2-q13 (Levan et al, 2006) |  | |  |  |  |  |  |  |
| **20q** | **↓** |  |  |  |  |  |  | 20:q13.31 (CSTF1, #770) |  |  | ↓ at 20q: 20q13.1-13.2 (Kiechle *et al*, 2000) |  |  |  |  |  | |  |  |  |  |  |  |
| **21q** | **↑** |  |  |  |  | SCC: 21:q21.3 (SCCT8, #664) |  |  |  |  |  |  |  | ↑/↓ at 21q |  |  | |  |  |  |  |  |  |
|  | **↓** |  |  |  |  | Gu,Gs: 21:q21.3 (SCCT8, #664) |  |  |  |  |  | ↓ at 21q in microsomally unstable (Suzuki *et al*, 1997), ↓ at 21q (Schulten *et al*, 2004) |  | ↑/↓ at 21q |  |  | |  |  |  |  |  |  |
| a | b | c | | | d | | e | f | g | h | i | j | k | l | m | n | | o | p | q | r | s | t |
| **22q** | **↑** |  |  |  | SCC: 22:q11.21 (COMT, #1265) |  |  |  |  |  |  |  | ↑ at 22q | ↑/↓ at 22q |  |  | |  |  |  |  |  |  |
|  | **↓** |  |  |  | Gu,Gs: 22:q11.21 (COMT, #1265) |  |  |  | 22:q13.2 (XRSCC6, #527) | ↓ at 22q13.2-13.3 |  | ↓ at 22q (Schulten *et al*, 2004) |  | ↑/↓ at 22q |  |  | |  |  |  |  |  |  |
| **Xq** | **↑** |  |  |  |  |  | Gs: X:q11.1 (MSN, #515) | Gu,Gs: X:q28 (EMD, #1150) |  |  |  | ↑/↓ at X (Sonoda*et al*, 1997), ↑ at Xq in microsomally unstable (Suzuki *et al*, 1997) |  | ↓/↑ at X |  |  | |  |  |  |  |  |  |
|  | **↓** |  |  |  |  |  | SCC. X:q11.1 (MSN, #515) | SCC: X:q28 (EMD, #1150) |  |  |  | ↑/↓ at X (Sonoda *et al*, 1997), ↓ at Xq - in stable (Suzuki *et al*, 1997) | from CIN2: ↓ at X | ↓/↑ at X |  |  | |  |  |  |  |  |  |

Note:

a – chromosome,

b - gains on/of chromosomes are labeled as ↑ and losses as ↓,

c – proteins over-expressed in genomically unstable EEC (Gu) and in SCC,

d - proteins under-expressed in genomically unstable EEC (Gu) but over-expressed in SCC,

e - proteins under-expressed in genomically unstable EEC (Gu) and in SCC,

f - proteins under-expressed in SCC,

g - proteins under-expressed in genomically unstable EEC (Gu) and genomically stable EEC (Gs) and in SCC,

h – chromosomal changes in the atypical hyperplasia of endometrium (AH),

i - chromosomal changes during the transfer of the complex hyperplasia of endometrium (CH) to AH and EEC,

j - chromosomal changes in EEC,

k - chromosomal changes during the transfer of the cervical intraepithelial neoplasia grade 3 (CIN 3) into the stage 1A of SCC,

l - chromosomal changes in SCC of stage IIB-IV,

m – t: chromosomal changes related to worse prognosis for patients with EEC (m), high metastatic potential of EEC (n), low differentiation (G3) of EEC (o), moderate differentiation (G2) of EEC (p), stage I (q), stage II (r), stage III (s), stage IV (t) of EEC.
